# Decoding seasonal changes: soil parameters and microbial communities in tropical dry deciduous forests

**DOI:** 10.3389/fmicb.2024.1258934

**Published:** 2024-02-19

**Authors:** Anjali Chandrol Solanki, Narendra Singh Gurjar, Satish Sharma, Zhen Wang, Ajay Kumar, Manoj Kumar Solanki, Praveen Kumar Divvela, Kajal Yadav, Brijendra Kumar Kashyap

**Affiliations:** ^1^Department of Agriculture, Mansarover Global University, Bhopal, Madhya Pradesh, India; ^2^Department of Soil Science and Agriculture Chemistry, Rajmata Vijayaraje Scindia Krishi Vishwa Vidyalaya, Gwalior, Madhya Pradesh, India; ^3^Department of Plant Pathology, B. M. College of Agriculture, Khandwa, Madhya Pradesh, India; ^4^Guangxi Key Laboratory of Agricultural Resources Chemistry and Biotechnology, Agricultural College, Yulin Normal University, Yulin, China; ^5^Amity Institute of Biotechnology, Amity University, Noida, Uttar Pradesh, India; ^6^Department of Life Sciences and Biological Sciences, IES University, Bhopal, Madhya Pradesh, India; ^7^Plant Cytogenetics and Molecular Biology Group, Institute of Biology, Biotechnology and Environmental Protection, Faculty of Natural Sciences, University of Silesia in Katowice, Katowice, Poland; ^8^Contec Global Agro Limited, Abuja, Nigeria; ^9^Department of Biotechnology, All India Institute of Medical Sciences, New Delhi, India; ^10^Department of Biotechnology Engineering, Institute of Engineering and Technology, Bundelkhand University, Jhansi, Uttar Pradesh, India

**Keywords:** seasonal variation, forest soil, soil parameters, bacteria, fungi

## Abstract

In dry deciduous tropical forests, both seasons (winter and summer) offer habitats that are essential ecologically. How these seasonal changes affect soil properties and microbial communities is not yet fully understood. This study aimed to investigate the influence of seasonal fluctuations on soil characteristics and microbial populations. The soil moisture content dramatically increases in the summer. However, the soil pH only gradually shifts from acidic to slightly neutral. During the summer, electrical conductivity (EC) values range from 0.62 to 1.03 ds m^-1^, in contrast to their decline in the winter. The levels of soil macronutrients and micronutrients increase during the summer, as does the quantity of soil organic carbon (SOC). A two-way ANOVA analysis reveals limited impacts of seasonal fluctuations and specific geographic locations on the amounts of accessible nitrogen (N) and phosphorus (P). Moreover, dehydrogenase, nitrate reductase, and urease activities rise in the summer, while chitinase, protease, and acid phosphatase activities are more pronounced in the winter. The soil microbes were identified in both seasons through 16S rRNA and ITS (Internal Transcribed Spacer) gene sequencing. Results revealed Proteobacteria and Ascomycota as predominant bacterial and fungal phyla. However, *Bacillus, Pseudomonas*, and *Burkholderia* are dominant bacterial genera, and *Aspergillus, Alternaria,* and *Trichoderma* are dominant fungal genera in the forest soil samples. Dominant bacterial and fungal genera may play a role in essential ecosystem services such as soil health management and nutrient cycling. In both seasons, clear relationships exist between soil properties, including pH, moisture, iron (Fe), zinc (Zn), and microbial diversity. Enzymatic activities and microbial shift relate positively with soil parameters. This study highlights robust soil-microbial interactions that persist mainly in the top layers of tropical dry deciduous forests in the summer and winter seasons. It provides insights into the responses of soil-microbial communities to seasonal changes, advancing our understanding of ecosystem dynamics and biodiversity preservation.

## 1 Introduction

Forests, covering 31% of Earth’s land area, harbor rich biodiversity, which is crucial for energy, water, and nutrient cycles (Keenan et al., 2015; FAO and UNEP, 2020). In addition, they act as the primary source of carbon sequestration on Earth, regulating climate change impact through microbial activity (García-Palacios and Chen, 2022; Baldrian et al., 2023). As the tropical forests, renowned for biodiversity, store 40% of global carbon stocks (Phillips et al., 1998; Kothandaraman et al., 2020), India boasts an expansive forested area, covering approximately 69.1 million hectares, ranking it as the world’s 10th largest forest region. Tropical dry deciduous forests cover the highest percentage, and they are more than one-third of the total forests (Joshi et al., 2010; Keenan et al., 2015; Reddy et al., 2015). Moreover, the forest of Madhya Pradesh, a state of India, is occupied predominantly by tropical dry deciduous forests (Raha et al., 2020), and these forests offer various agricultural benefits like climate influence, timber resources, soil fertility maintenance, water retention, and carbon sequestration, enhancing environmental sustainability and resilience in arid regions. Tropical ecosystems are important in maintaining biodiversity and regulating carbon emissions (Joshi and Dhyani, 2019; Khosravi Mashizi and Sharafatmandrad, 2023). These ecosystems act as vital carbon sinks, actively sequestering atmospheric carbon dioxide and contributing to the global effort to mitigate climate change. In addition, these forests provide homes for various wildlife, including endangered tigers (Banerjee et al., 2020; Sarma and Barpujari, 2023). Tropical habitats are essential managers of biodiversity, crucial for preserving various plant and animal species. Thus, the conservation and management of tropical forests are vital for biodiversity conservation and their essential role in carbon sequestration.

In tropical forests, soil fungi and bacteria are the key players in transforming and processing carbon and nutrients through microbial processes (Camenzind et al., 2018). However, despite the substantial coverage of tropical dry deciduous forests in Madhya Pradesh, data on soil biochemical properties and microbial activity remain limited, impeding our ability to assess soil health and related biological processes. Unfortunately, few studies have surveyed these regions to determine biomass reserves and forest soil productivity (Pande, 2005; Salunkhe et al., 2014; Raha et al., 2020). Insufficient information on soil health in the tropical dry deciduous forests of Madhya Pradesh poses a threat to agricultural output, ecological services, biodiversity, and effective conservation. Strategies for mitigating climate change are hampered by incomplete understanding of the carbon cycle and biodiversity threats.

Seasonal variations in tropical dry deciduous forests, such as rainfall precipitation and temperature changes, can impact soil nutrients, microbial activities, and plant performance. Previously, Chaturvedi et al. (2011) reported that in tropical dry deciduous forests, environmental variables like temperature and rainfall can impact soil nutrients and microbial activity in the soil ecosystem, influencing plant fitness. For example, in *Pinus pinaster* forests, soil fungi exhibit specific responses to variations in moisture and temperature, with mycorrhizal fungi displaying greater resilience to summer drought conditions (Castaño et al., 2018). Alterations in litter and root inputs further influence soil carbon content and microbial populations, with the addition of litter significantly boosting both organic carbon and microbial biomass (Feng et al., 2022). Seasonal changes in environmental factors such as summer or winter and soil fertility have been found to impact soil parameters and microbial communities in tropical dry deciduous forests (Pajares et al., 2018). The spatiotemporal variation of soil microbial communities in tropical forests is mainly explained by regional location-induced changes in climatic factors and edaphic properties (Wei et al., 2022). Soil nutrients and enzymatic activity serve as vital indicators of soil health, ensuring vegetation sustainability and maintaining balanced ecosystems, especially in agricultural lands (Alkorta et al., 2003; Page-Dumroese et al., 2021). In the ecosystems of forests, various microbial species play crucial functions in the soil process and dynamics (Baldrian, 2017b; Deng et al., 2022). Within forest soils, microorganisms play a pivotal role in breaking down complex organic matter and recycling essential nutrients, ultimately supporting soil fertility and facilitating plant growth (Krishna and Mohan, 2017; Chacon et al., 2023; Li et al., 2023; Lv et al., 2023; Zhou et al., 2023). For instance, *Cellulomonas* sp. is responsible for the breakdown of cellulose (Ogola et al., 2021), while *Rhizobium* spp. need to establish a symbiotic relationship with leguminous plants to fix nitrogen (Goyal and Habtewold, 2023). *Bacillus* spp. aid in the solubilization of phosphorus (Mason et al., 2021; Shang et al., 2021), *Pseudomonas* spp. play a crucial role in decomposing organic matter (Bastida et al., 2021; Shang et al., 2021), and *Streptomyces* spp. regulate the activity of other microorganisms (Grosso et al., 2018). Previous studies revealed saprophytic fungi, major decomposers in the soil, secrete soil oxidation enzymes during the decomposition of organic materials (Wang L. et al., 2023). For example, mycorrhizal fungi such as *Glomus* spp. enhance the absorption of nutrients, *Agaricus* spp. facilitate the recycling of organic material, and lignin-degrading fungi like *Trichoderma* play a critical role in carbon cycling (Jasu et al., 2021). These microbial reactions are indispensable for acquiring carbon, nitrogen, and phosphorus. Furthermore, oxidoreductases contribute to the bio-composting of organic compounds (Tiemann and Billings, 2010). These far-reaching microbial reactions are essential for ensuring a productive food system and improving plant health (Singh and Trivedi, 2017). In forest soil, microbial communities possess defined functions in the degradation of plant biomass and composting (Baldrian, 2017a; Zhang et al., 2022; Sardar et al., 2023).

Soil microorganisms secrete extracellular enzymes while decomposing organic materials (Burns et al., 2013; Liu et al., 2023). These enzymes not only serve as indicators of soil health but also act as crucial catalysts that regulate biological activities in the plant rhizosphere, influencing the development of microbial communities (Wang et al., 2015; Li et al., 2017). However, this process is essential for maintaining soil fertility and supporting plant growth (Hartmann and Six, 2023). This interplay between soil cycling and enzyme dynamics creates a harmonious environment that fosters the sustained development of various plant species and microorganisms. Moreover, this process influences the composition and activities of microbial communities, further contributing to the overall health and resilience of the ecosystem.

Previous studies highlight the influence of environmental variables on soil nutrients and microbial activity, impacting overall plant fitness. However, a more in-depth exploration is needed to unravel the intricate relationships between soil attributes, microbial populations, and seasonal fluctuations in Madhya Pradesh’s tropical dry deciduous forests. Consequently, the present study aims to achieve the following objectives: (1) Assess nutrient levels in the top (organic) layer of soil across three different forest areas during two distinct seasons, (2) isolate and identify the bacterial and fungal communities, and (3) understand the relationship between soil biochemical parameters and the composition of microbial taxa in the tropical dry deciduous forests of Madhya Pradesh. These objectives collectively contribute to a holistic understanding of the soil-microbe interactions in the studied ecosystem. This knowledge is crucial for maintaining soil fertility, supporting plant growth, and ensuring the long-term health of these forests.

## 2 Materials and methods

### 2.1 Study area and description

The seasonal variation experiment was conducted in a tropical dry deciduous forest in three districts of Madhya Pradesh, India: Sehore, Hoshangabad, and Betul. Geographically, the sampling sites were situated between 21°11°–22°88΄ N and 77°08΄ –78°24΄E, covering the Malva Plateau (MP: near Kotara village), Narmada Valley (NV: near Barandua village), and Satpura Valley (SP: near Bakud village) regions (Supplementary Table S1), and their diverse environmental conditions (Supplementary Table S2). These districts were selected to represent a diverse range of environmental conditions, including variations in soil fertility, forest composition, wildlife presence, landscape characteristics, rainfall patterns, human activities, and soil types within the tropical dry deciduous forests of Madhya Pradesh. This deliberate selection allowed for a comprehensive exploration of the intricate interplay among these factors and their influence on the sustainability and dynamics of these crucial ecosystems. This forest area, situated at an elevation of 300–500 m a.s.l., covers approximately 88.65% of the total forested region and has remained relatively undisturbed by direct human activities for an extended period. However, it is moderately susceptible to fires. The dominant plant species in the area include *Tectona grandis, Shorea robusta, Lagerstroemia parviflora, Diospyros melanoxylon*, and *Anogeissus latifolia*. However, Madhya Pradesh experiences a subtropical climate with distinct seasons, including dry summers from April to June, monsoon season from July to September, and winter from October to February. During summer, average maximum and minimum temperatures are approximately 41.67 ± 1.53 and 37.00 ± 1.00°C, respectively, while in winter, they are around 26.67 ± 1.53 and 13.00 ± 1.00°C. Annual average rainfall in these regions ranges between 850 and 950 mm, with nearly 85% occurring during the monsoon season and the remaining 15% distributed between summer and winter. The surface forest soils are typical black soil with medium fertility, primarily composed of basaltic rocks, and contain significant proportions of iron and lime rocks.

### 2.2 Soil sampling

Three naturally established forest areas were chosen (Supplementary Figure S1, Supplementary Table S1), and soil samples from the forest surface were collected during two different seasons over 1 year. The selection of these three distinct forest areas aimed to capture a comprehensive range of ecological conditions within the study region, enabling a robust understanding of the effects of seasonal variations on soil parameters and microbial communities. The first sampling was conducted in May 2021 (during summer), and the second in November 2021 (during winter). After removing surface litter, 10–12 soil cores in triplicate, each with a diameter of 4 cm, were collected from the organic horizon (0–15 cm depth) of each plot (10 m × 10 m). Each site comprises three plots, with a minimum separation of 20 meters between each plot, designated for sampling. Soil samples were collected at three random points within each plot. The organic horizon (0–15 cm depth) was chosen as the target area for soil sampling due to its significance as a region of increased biological and chemical activity, providing insightful information about the dynamic interactions between soil components and microorganisms vital to ecosystem function. The collected cores were composited based on plot and depth, placed in plastic bags, stored in coolers, and transported to the laboratory. There, they were refrigerated for 1–2 weeks before sample processing and analysis. Any unwanted impurities, such as stones, gravel, and forest litter, were carefully removed from the samples. These soil samples were then utilized to analyze soil physicochemical properties, soil microorganisms, and enzymatic activities. Soil samples were collected from the exact locations in both seasons (summer and winter) to ensure consistency.

### 2.3 Soil biochemical analysis

The soil pH and electrical conductivity were determined in a 1:2 soil-to-water suspension after allowing the soil samples to stand overnight following the standard protocol of Piper (1950). Soil organic carbon (SOC) analysis was performed using Walkley and Black’s wet oxidation method (Walkley and Black, 1934). The gravimetric method estimated Soil moisture content using a hot air oven (Misra, 1968). Available soil nitrogen was quantified using the alkaline permanganate method (Subbiah and Asija, 1956). Soil-available phosphorus was assessed using Olsen’s extraction procedure (Olsen, 1954), with 0.5 M NaHCO_3_ (pH 8.5), and determination was carried out using the ascorbic acid method (Bremner and Mulvaney, 1982). Soil-available potassium was extracted using ammonium acetate and measured with a flame photometer (Systronics-128; Muhr et al., 1965). To determine micronutrient cations (Cu, Fe, Mn, and Zn), soil samples were extracted with 0.005 M diethylene triamine pentaacetic acid (DTPA) and analyzed using Atomic Absorption Spectrophotometer (Perkin Elmer 3,110; Lindsay and Norvell, 1978).

### 2.4 Soil enzyme activities

The present study assessed six soil enzymes: chitinase, dehydrogenase, protease, acid phosphatase, nitrate reductase, and urease. Chitinase activity was measured using the acetylene reduction assay (ARA) and expressed as μg g^−1^ soil (Trotta et al., 1996), playing an essential role in the breakdown of organic materials. Soil quality and microbial activity indicator enzyme dehydrogenase were determined using triphenyl tetrazolium chloride (TTC) as a substrate (Lenhard, 1956; Thalmann, 1968) and expressed as μg TPF g^−1^ soil h^−1^. Protease activities were measured using the ninhydrin colorimetric method described by Guan et al. (1986) and defined as mg g^−1^ soil 24 h^−1^ because this enzyme affects plant nutrient availability and nitrogen cycling. Acid phosphatase activity, responsible for the mineralization of phosphorus, was estimated using р-nitrophenyl phosphate (р-NPP) as a substrate as described by Schneider et al. (2008) and expressed as μg р-NPP g^−1^ 24 h^−1^. Nitrite reductase activity was examined using the Gerry reagent method and expressed as mg NO_2_-N g^−1^ 24 h^−1^ to understand nitrogen transformations (Li et al., 2008). Urease activity, to know nitrogen mineralization, was assessed via phenol-sodium hypochlorite calorimetry and described as mg NH_3_-N kg^−1^ 3 h^−1^ (Guan et al., 1986). All activities were determined at 30°C and ambient soil pH without buffers. Each experiment was performed in triplicate.

### 2.5 Soil microbial community analysis

For the microbial communities analysis, bacterial strains were isolated by standard serial dilution method using nutrient agar (NA; HiMedia, Mumbai, India) and tryptic soy agar (TSA; HiMedia, Mumbai, India) plates. In brief, soil samples (10 g) were incubated in 90 mL of sterile saline water shaken at room temperature (150 rpm) for 2 h. Subsequently, 100 μL aliquots of serial dilutions were plated on NA and TSA plates with nystatin (50 μg mL^−1^) to inhibit fungal growth and then incubated at 37°C for 24–48 h. Distinct bacterial colonies were selected and streaked on fresh culture media to obtain pure cultures.

Similarly, for fungal isolation, 100 μL aliquots of serial dilutions were plated on potato dextrose agar medium (PDA; HiMedia, Mumbai, India) and Sabouraud dextrose agar medium (SDA; HiMedia, Mumbai, India) with chloramphenicol (20 μg mL^−1^) and streptomycin sulfate (50 μg mL^−1^) to inhibit bacterial growth. Plates were incubated at 28°C for 48–72 h, and phenotypically distinct fungal colonies were transferred to PDA plates and subcultured twice for pure cultures. Isolated bacterial and fungal cultures were stored as agar slants and frozen at −80°C in 20% glycerol. DNA extraction from bacteria and fungi was performed using a DNA isolation Kit (HiMedia, Mumbai, India). The bacterial 16S rRNA gene and fungal ITS gene region were amplified using universal primers 27F/1492R and ITS1/ITS4, respectively (Supplementary Table S3). PCR products were purified and sequenced via standard Sanger sequencing using the ABI3100 Genetic Analyzer with respective primers. The sequencing reaction employed the Big Dye Terminator Cycle Sequencing Kit V3.1 (Applied Biosystems, United States) following the manufacturer’s protocol. The resulting sequence chromatograms were analyzed using DNASTAR Lasergene sequence analysis software (Burland, 2000), to generate sequences for molecular identification. Low-quality regions were trimmed, and ambiguous bases were removed. Isolate sequences were aligned and classified with SINA 1.2.11, utilizing the bacterial small subunit (SSU) in SILVA 115NR99 (Quast et al., 2013) and UNITE (Unified System for the DNA Based Fungal Species Linked to the Classification Outline) for fungi. The obtained sequences were aligned with the NCBI GenBank database for taxonomic identification up to the species level. After taxonomic distribution identification, a Sankey diagram was created, and identified sequences were submitted to NCBI GenBank. Microbial isolates’ distribution and the number of species in each location were recorded. The Past3 software was then employed to calculate species richness and diversity indices.

### 2.6 Statistical analysis

Data processing and statistical analyses were performed using SPSS IBM version 25 and Origin 2017SR2 software. The Least Significance Difference Test (LSD) at *p* < 0.05 significance level was utilized for *post-hoc* comparisons, providing insights into specific differences among treatments. Two-way ANOVA allowed us to explore interactions between two independent variables, unveiling the combined effects of seasons and locations on soil parameters. Regression analysis, conducted in Microsoft Excel Office 2013, helped establish relationships between microbial diversity and soil factors. Correlation analysis, executed in Past3 software, facilitated an understanding of associations between variables. A heatmap, generated with TB tools, visually represented patterns in data, aiding in identifying trends and connections within the dataset.

## 3 Results

### 3.1 Soil physicochemical properties

#### 3.1.1 Seasonal and location variances

The investigation into soil physicochemical properties revealed distinctive patterns among the studied locations. NV consistently exhibited higher pH, moisture, EC, and SOC values during both summer and winter seasons. Similarly, SP soil samples demonstrated elevated levels of available N and P content, while MP showed a higher quantity of available K in both seasons (Table 1). The pH of forest soils across all locations ranged from 6.5 to 7.6 during the summer, indicating a slightly neutral condition, whereas it became moderately acidic (pH 5.5–6.5) during winter (Table 1). Furthermore, the moisture content was notably higher in summer (39 to 45%) compared to winter (25 to 31%). The EC value ranged from 0.62 to 1.03 ds m^−1^ in summer and decreased during winter (0.35 to 0.89 ds m^−1^). SOC content and available N, P, and K displayed higher values in summer than in winter (Table 2). Soil micronutrients increased during the summer, including Cu, Fe, Mn, and Zn. Significant quantities of Fe, Mn, and Zn values were mainly found in SP samples during both seasons, while significantly (*p* < 0.05) higher Cu values were observed in NV samples (Table 2). The factorial ANOVA results emphasized the significant influence of both seasons and locations on soil properties (Table 3). Notably, available nitrogen and available phosphorus showed a less significant (*p* < 0.05) response to seasonal changes compared to other parameters. Regarding location, SOC, available N, and available P demonstrated a less significant (*p* < 0.05) response to seasonal changes than others. The interaction between seasons and locations (S x L) significantly impacted soil properties, except for SOC, nitrogen, phosphorus, potassium, soil pH, and moisture. EC showed a less significance (*p* < 0.05) response to seasonal changes compared to micronutrients (Cu, Fe, Mn, and Zn; Table 3).

**Table 1:** Seasonal variation on soil physicochemical properties of tropical dry deciduous forest.

Locations	Seasons	pH	Moisture (%)	EC (ds m^−1^)	SOC (g kg^−1^ soil)
MP	Summer	7.20 ± 0.14^b^	41.60 ± 0.92^b^	0.62 ± 0.04^d^	7.16 ± 0.15^b^
	Winter	5.83 ± 0.04^d^	31.07 ± 1.48^c^	0.35 ± 0.03^e^	5.79 ± 0.83^c^
NV	Summer	7.68 ± 0.08^a^	45.16 ± 0.54^a^	1.03 ± 0.04^a^	8.11 ± 0.43^a^
	Winter	6.72 ± 0.18^c^	31.83 ± 0.42^c^	0.89 ± 0.02^b^	5.92 ± 0.56^c^
SP	Summer	6.55 ± 0.11^c^	39.90 ± 1.61^b^	0.82 ± 0.03^c^	7.00 ± 0.28^b^
	Winter	5.48 ± 0.02^e^	25.53 ± 0.71^d^	0.61 ± 0.06^d^	4.48 ± 0.12^d^
SED		0.09	0.86	0.03	0.38
CD		0.19	1.87	0.07	0.83
CV		1.60	2.90	5.30	7.30

MP, Malva plateau; NV, Narmada valley; and SP, Satpura valley; CD, critical difference; SED, Standard error of difference; CV, Coefficient of variation. Averaged values (*n* = 3) within a column, succeeded by different small letters, differ significantly at *p* < 0.05 significance level through the LSD test.

**Table 2:** Seasonal variation on soil macro and micronutrients of tropical dry deciduous forest.

Locations	Seasons	N (kg ha^−1^)	P (kg ha^−1^)	K (kg ha^−1^)	Cu(ppm)	Fe(ppm)	Mn(ppm)	Zn(ppm)
MP	Summer	144.93 ± 18.34^ab^	17.99 ± 1.07^bc^	32.19 ± 1.79^a^	1.57 ± 0.12^c^	12.19 ± 0.15^c^	12.61 ± 0.33^c^	0.32 ± 0.02^d^
	Winter	132.14 ± 17.01^ab^	16.90 ± 0.54^c^	29.04 ± 0.83^b^	1.35 ± 0.01^d^	9.73 ± 0.03^e^	10.05 ± 0.04^d^	0.20 ± 0.01^e^
NV	Summer	138.33 ± 9.42^ab^	18.35 ± 1.67^bc^	26.94 ± 0.60^bc^	7.65 ± 0.05^a^	10.51 ± 0.41^d^	12.72 ± 0.45^c^	0.49 ± 0.02^c^
	Winter	125.77 ± 8.56^b^	16.65 ± 1.51^c^	25.40 ± 0.54^c^	6.48 ± 0.04^b^	7.92 ± 0.00^f^	10.65 ± 0.04_d_	0.36 ± 0.01^d^
SP	Summer	160.02 ± 27.71^a^	20.43 ± 0.37^a^	32.10 ± 1.42^a^	1.24 ± 0.04^d^	50.70 ± 0.30^a^	28.69 ± 0.12^a^	1.47 ± 0.04^a^
	Winter	145.49 ± 25.17^ab^	18.90 ± 0.19^ab^	28.72 ± 1.48^b^	0.89 ± 0.00^e^	43.45 ± 0.18^b^	23.89 ± 0.05^b^	0.86 ± 0.00^b^
SED		15.60	0.86	0.99	0.08	0.32	0.33	0.03
CD		33.98	1.88	2.15	0.18	0.71	0.72	0.07
CV		13.50	5.80	4.20	3.20	1.80	2.50	6.00

MP, Malva plateau; NV, Narmada valley; and SP, Satpura valley; CD, critical difference; SED, Standard error of difference; CV, Coefficient of variation. Averaged values (*n* = 3) within a column, succeeded by different small letters, differ significantly at *p* < 0.05 significance level through the LSD test.

**Table 3:** Summary of two-way ANOVA for the effects of two seasons (s) and three locations (L) on soil properties.

	Season (S)		Locations (L)		S × L	
Parameters	DF (−1)		(DF-2)		(DF-2)	
	*F*	*P*	*F*	*P*	*F*	*P*
pH	490.20	***	180.80	***	5.90	*
Moisture	664.30	***	46.51	***	5.36	*
Electrical Conductivity	128.20	***	230.90	***	4.32	*
Soil Organic Carbon	85.10	***	11.29	**	2.39	0.14
Nitrogen	8.49	*	8.91	**	0.04	0.96
Phosphorus	8.35	*	8.63	**	0.13	0.88
Potassium	22.33	***	25.97	***	1.03	0.39
Copper	143.20	***	6,324	***	36.83	***
Iron	479.50	***	17400.00	***	70.91	***
Manganese	270.10	***	2657.00	***	19.26	***
Zinc	271.40	***	992.90	***	87.86	***

Significance level: *p < 0.05; **p < 0.01; ***p < 0.001; DF, degrees of freedom.

#### 3.1.2 Soil enzyme activity

Soil enzyme activity revealed distinct patterns in NV, SP, and MP soils. NV exhibited higher dehydrogenase, protease, acid phosphatase, and nitrate reductase activities, while SP showed higher urease activity. Additionally, SP displayed higher chitinase activity during the summer, while MP exhibited higher chitinase activity in winter (Table 4), and higher chitinase activity resulted in winter that may enhance the decomposition of organic matter. Notably, dehydrogenase, nitrate reductase, and urease enzyme activities increased in the summer, whereas chitinase, protease, and acid phosphatase enzyme activities were higher during the winter (Table 4). Two-way ANOVA results indicated that seasonal variations significantly impacted enzymes, with locations playing a secondary role. The interaction between S x L showed a significant interactive effect with chitinase, dehydrogenase, protease, acid phosphatase, and nitrate reductase, while urease showed an interactive non-significant response (Table 5).

**Table 4:** Seasonal dynamics of soil enzymatic activities of tropical dry deciduous forest.

Locations	Seasons	Chitinase	Dehydrogenase	Protease	Acid phosphatase	Nitrate reductase	Urease
		μg g^−1^ soil	μg TPF g^−1^soil h^−1^	mg g^−1^ soil 24 h^−1^	μg р-NPP g^−1^ 24 h^−1^	mg NO_2_-N g^−1^ 24 h^−1^	NH_3_-N kg^−1^ 3 h^−1^
MP	Summer	2.99 ± 0.21^d^	12.73 ± 0.06^c^	150.24 ± 0.82^d^	32.95 ± 1.16^c^	68.87 ± 0.70^b^	7.42 ± 0.39^c^
	Winter	4.19 ± 0.16^a^	11.07 ± 0.03^e^	189.37 ± 3.60^b^	38.69 ± 0.97^b^	53.67 ± 6.52^d^	5.83 ± 0.20^d^
NV	Summer	2.48 ± 0.22^e^	14.99 ± 0.21^a^	162.70 ± 2.44^c^	38.10 ± 1.28^b^	76.87 ± 5.05^a^	8.75 ± 0.50^b^
	Winter	3.59 ± 0.09^b^	12.36 ± 0.43^cd^	195.31 ± 1.07^a^	42.44 ± 1.02^a^	64.13 ± 1.03^bc^	7.40 ± 1.26^c^
SP	Summer	3.31 ± 0.04^c^	13.51 ± 0.35^b^	145.39 ± 0.80^d^	21.23 ± 0.99^d^	65.57 ± 0.46^bc^	10.18 ± 0.28^a^
	Winter	3.86 ± 0.14^b^	11.91 ± 0.60^d^	167.36 ± 5.34^c^	32.80 ± 1.68^c^	61.03 ± 1.72^c^	7.88 ± 0.42^bc^
SED		0.13	0.28	2.35	0.99	2.84	0.51
CD		0.28	0.61	5.13	2.15	6.19	1.10
CV		4.60	2.70	1.70	3.50	5.40	7.80

MP, Malva plateau; NV, Narmada valley; and SP, Satpura valley; CD, critical difference; SED, Standard error of difference; CV, Coefficient of variation. Averaged values (*n* = 3) within a column, succeeded by different small letters, differ significantly at *p* < 0.05 significance level through the LSD test.

**Table 5:** Summary of two-way ANOVA for the effects of two seasons (S) and three locations (L) on soil enzymes.

		Chitinase	Dehydrogenase	Protease	Acid phosphatase	Nitrate reductase	Urease
Season (S) DF (−1)	F	168.60	146.00	528.60	159.80	43.48	36.07
	P	***	***	***	***	***	***
Locations (L) (DF-2)	F	24.82	39.69	93.64	186.30	11.65	23.03
	P	***	***	***	***	**	***
S × L (DF-2)	F	7.56	4.21	13.55	15.03	3.86	0.96
	P	**	*	**	**	0.05	0.40

Significance level: *p < 0.05; **p < 0.01; ***p < 0.001; DF, degrees of freedom.

### 3.2 Pearson’s correlation analysis

**Figure 1:**
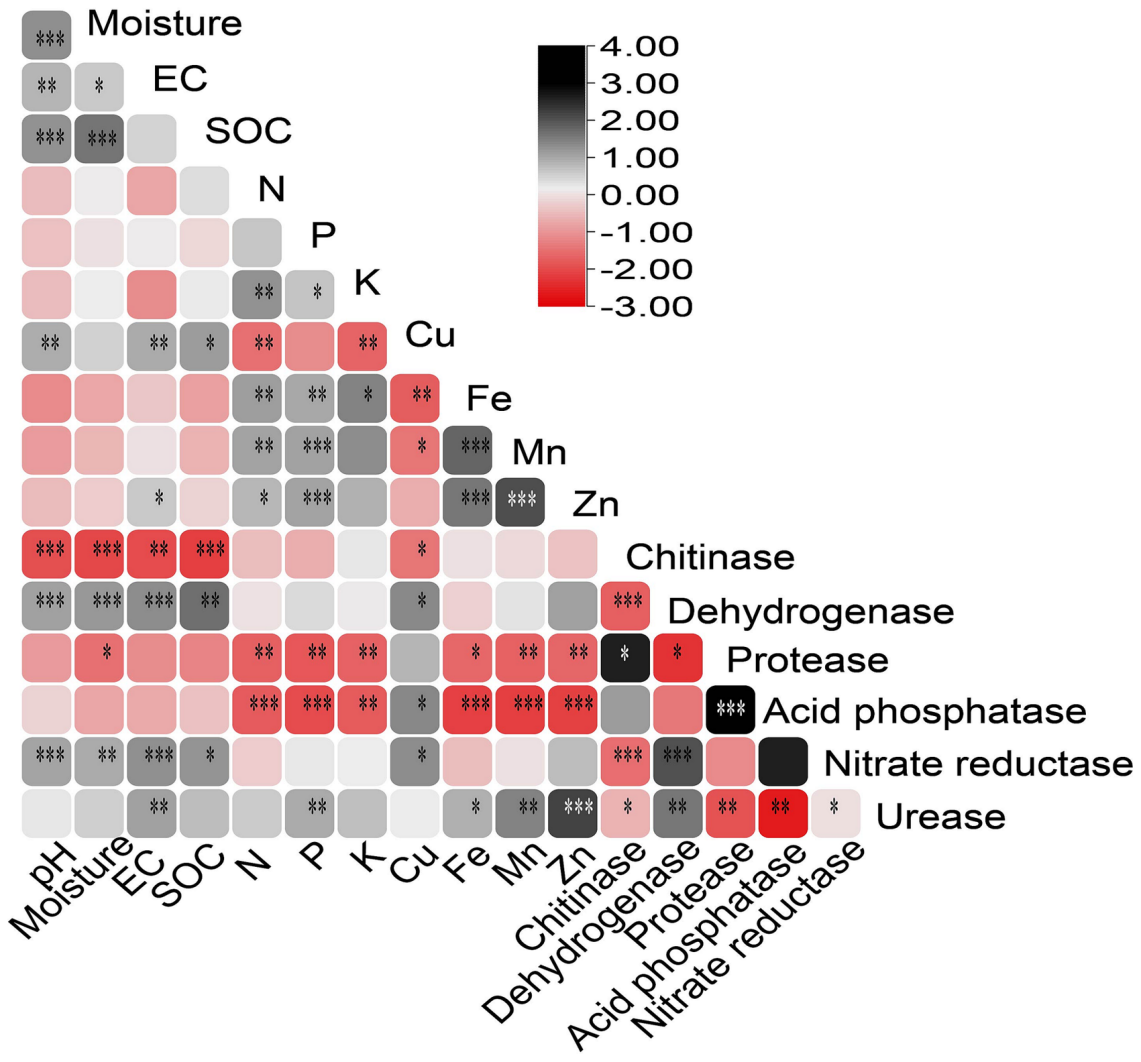
Pearson correlation matrix of forest soil parameters, including physiological, chemical, mineral, and enzyme data. EC, electric conductivity; SOC, soil organic carbon. (*p < 0.05; **p < 0.01; ***p < 0.001).

Pearson’s correlation analysis provided insights into the relationships between soil parameters (Figure 1). Soil pH significantly (*p* < 0.05) positively correlated with moisture, SOC, Cu, and EC. Soil moisture was positively associated with EC, SOC, and Cu. Significant (*p* < 0.05) positive correlations were observed among EC, SOC, and Cu. Soil N and P showed positive correlations with K, Fe, Mn, and Zn, but negative correlations with Cu. Soil K exhibited a positive correlation with Fe and a negative correlation with Cu. Moreover, significant positive correlations were observed between macronutrients Fe, Mn, and Zn. Regarding soil enzymes, chitinase activity showed a significant negative correlation (*p* < 0.05) with pH, moisture, EC, SOC, Cu, dehydrogenase, and nitrate reductase. Dehydrogenase and nitrate reductase positively correlated with pH, moisture, EC, SOC, Cu, and chitinase. Soil enzymes protease and acid phosphatase were negatively correlated with N, P, K, Fe, Mn, Zn, and urease. In comparison, urease showed a highly significant positive correlation (*p* < 0.05) with EC, Fe, Mn, and Zn (Figure 1).

### 3.3 Microbial community analysis

#### 3.3.1 Microbial isolation

Across all locations and seasons, a total of 111 bacterial isolates and 81 fungal isolates were identified. During the winter, the range of bacterial isolates varied from 16 to 19, with SP having 16, MP having 17, and NV leading with 19 isolates. Similarly, the range of fungal isolates during winter varied from 12 to 15, with MP having 12, SP having 14, and NV having the highest count of 15. Transitioning to the summer season, bacterial isolates exhibited variability between 19 and 21, with both SP and MP at 19, while NV displayed the highest count of 21. In contrast, fungal isolates during summer consistently numbered between 13 and 14 across different locations. These findings emphasize the differences in microbial populations based on location and seasonal changes. Notably, NV consistently demonstrated higher counts, particularly in the summer.

#### 3.3.2 Diversity indices

The bacterial and fungal diversity indices varied across locations during both seasons (Supplementary Table S4). The Shannon-Wiener index and evenness of bacteria were highest in MP during summer and NV during winter. Species richness was higher in NV samples during both seasons. For fungi, the highest values of the Shannon-Wiener index and species richness were observed in NV samples during both seasons, with MP showing higher evenness during both seasons (Supplementary Table S4).

#### 3.3.3 Bacterial microbiota composition

**Figure 2:**
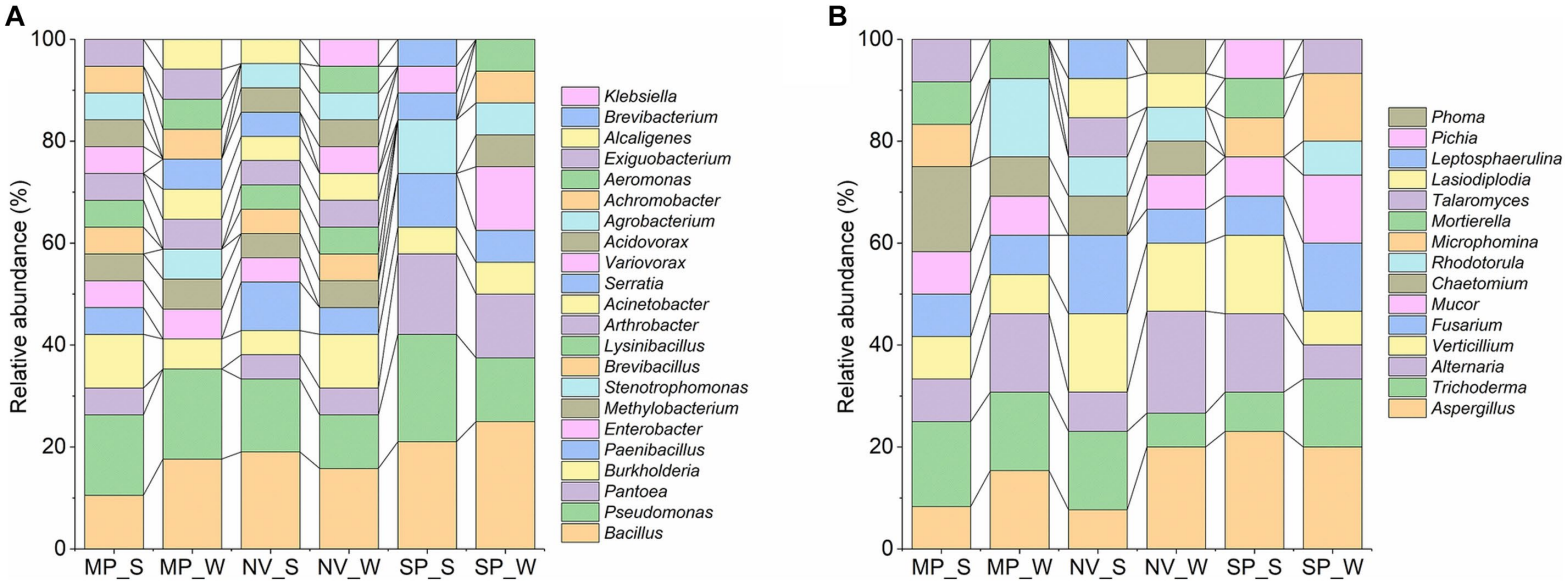
Relative abundance of bacterial **(A)** and fungal **(B)** genera in forest soil samples of MP, Malva plateau; NV, Narmada valley; and SP, Satpura valley during two seasons (S, Summer and W, Winter).

The results of 16S rRNA gene sequencing indicated that the overall bacterial microbiota of forest soil belonged to three phyla: Proteobacteria (62.16%), Firmicutes (33.33%), and Actinobacteria (4.50%). These were further distributed into four classes: γ-Proteobacteria (54.95%), α-Proteobacteria (7.21%), Bacilli (33.33%), and Actinobacteria (4.50%). Notably, Bacillales (21.62%), Pseudomonadales (18.02%), Enterobacterales (17.12%), and Burkholderiales (16.22%) were the main soil bacteria group orders. At the family level, dominant taxa included Bacillaceae (18.02%), Pseudomonadaceae (15.32%), Burkholderiaceae (7.21%), Erwiniaceae (7.21%), and Paenibacillaceae (6.31%). The top 10 bacterial genera were *Bacillus* (18.02%), *Pseudomonas* (15.32%), *Burkholderia* (7.21%), *Pantoea* (7.21%), *Paenibacillus* (6.31%), *Methylobacterium* (4.50%), *Enterobacter* (4.50%), *Arthrobacter* (3.60%), *Lysinibacillus* (3.60%), and *Brevibacillus* (3.60%; Figure 2A).

#### 3.3.4 Fungal microbiota composition

Regarding ITS region sequencing for fungi, the overall fungal microbiota belonged to three phyla: Ascomycota (82.72%), Mucoromycota (11.11%), and Basidiomycota (6.17%). These were further distributed into seven classes: Sordariomycetes (39.51%), Dothideomycetes (22.22%), Eurotiomycetes (19.75%), Mucoromycetes (7.41%), Microbotryomycetes (6.17%), Mortierellomycetes (3.70%), and Saccharomycetes (1.23%). The dominant soil fungi group orders were identified as Aspergillaceae (16.05%), Hypocreaceae (12.35%), Pleosporaceae (12.35%), Plectosphaerellaceae (11.11%), and Nectriaceae (9.88%). The top 10 fungal genera were *Aspergillus* (16.05%), *Alternaria* (12.35%), *Trichoderma* (12.35%), *Verticillium* (11.11%), *Fusarium* (9.88%), *Mucor* (7.41%), *Chaetomium* (6.17%), *Rhodotorula* (6.17%), *Microphomina* (4.94%), and *Talaromyces* (3.70%; Figure 2B).

#### 3.3.5 Microbial distribution

**Figure 3:**
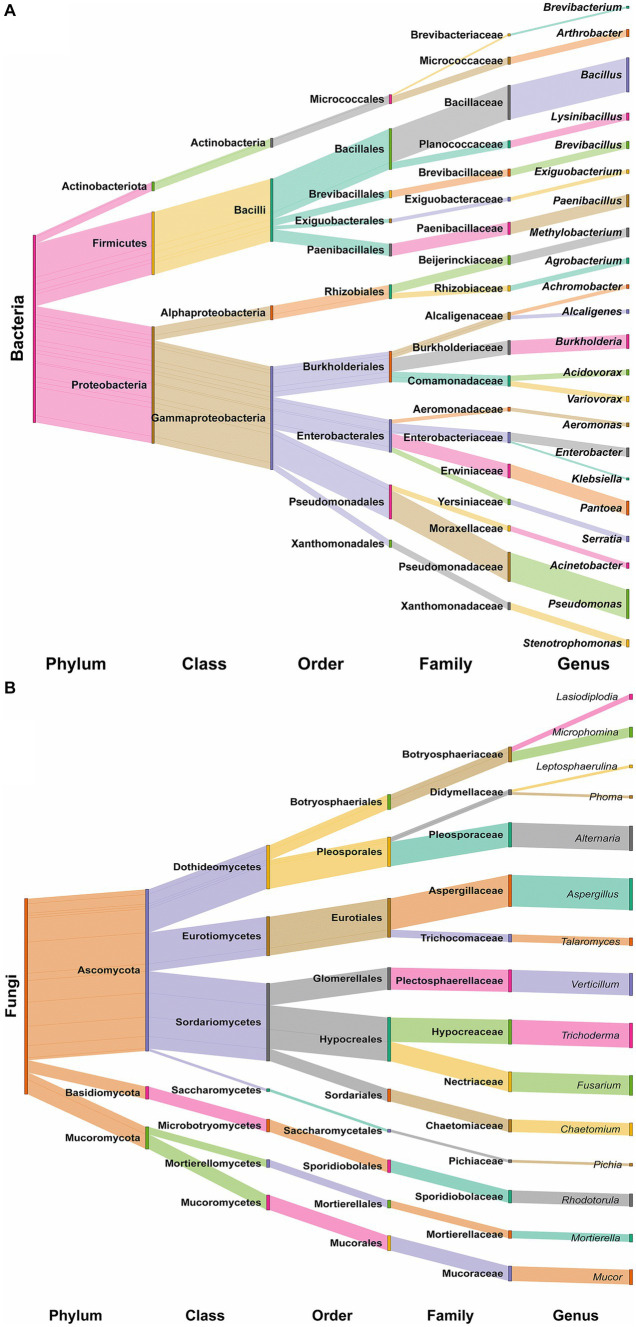
Sankey diagram showing the distribution of all identified bacterial taxa **(A)** and fungal taxa **(B)** at each taxonomic level.

The distribution and structure of the relative abundance of bacteria and fungi are represented in Figure 3. Notably, *Bacillus* and *Pseudomonas* in bacteria and *Aspergillus* in fungi exhibited season-specific variations in abundance across different locations. In terms of bacterial genera, *Bacillus* was found to be less abundant in summer in MP and SP, while it increased in NV soil samples during summer (Figure 3A). *Pseudomonas* exhibited higher abundance in NV and SP during summer and in MP samples during winter. The genera *Pantoea, Burkholderia, Paenibacillus, Enterobacter, Methylobacterium,* and *Stenotrophomonas* also showed location and season-specific variations (Figure 3A). As for fungi-dominant genera, *Aspergillus* was higher in winter in NV and MP samples and during summer in SP samples (Figure 3B). The specific genera and closely related species with NCBI accession numbers for bacteria and fungi, respectively, are described in Supplementary Tables S5, S6.

**Figure 4:**
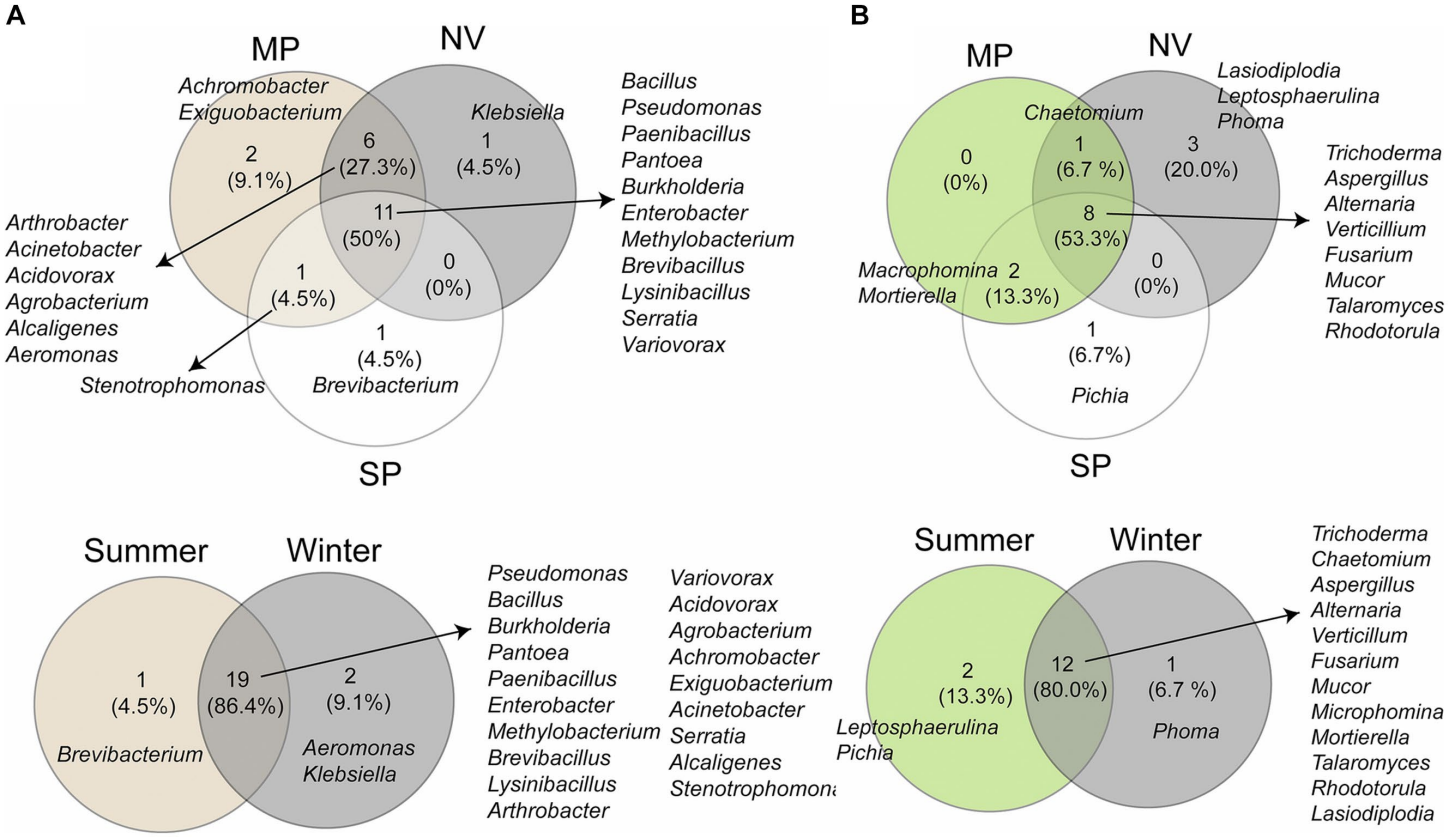
Venn diagrams illustrating the distribution of bacterial **(A)** and fungal **(B)** taxa among three locations (MP, Malva plateau; NV, Narmada valley; and SP, Satpura valley) and two seasons (summer and winter).

Furthermore, the Venn diagram revealed that 50% (11 genera) of bacterial genera were common to all locations, and 27.3% (6 genera) were shared between MP and NV samples (Figure 4A). The genus *Stenotrophomonas* was commonly present in both MP and SP samples. Additionally, two unique genera, namely *Achromobacter* and *Exiguobacterium*, were exclusively found in MP samples, while *Klebsiella* was exclusive to NV samples, and *Brevibacterium* was unique to SP samples. The Venn diagram also illustrated the structure of the bacterial community across both seasons (Figure 4A), with a total of 19 genera being common to samples from both seasons. Two unique genera, *Aeromonas* and *Klebsiella*, were identified in winter samples, and the genus *Brevibacterium* was present exclusively in winter samples.

As for the fungal microbiota (Figure 4B), the Venn diagram indicated that 53.3% of genera were shared among all locations, with 13.3% (2 genera) being common to both MP and SP samples. *Chaetomium* was found to be shared between MP and NV samples. Three unique genera, namely *Lasiodiplodia*, *Leptosphaerulina*, and *Phoma*, were exclusively found in NV samples, and the genus *Pichia* was unique to SP samples. However, 80% of fungal genera were shared between samples from both seasons. Two individual genera, *Leptosphaerulina* and *Pichia*, were identified in summer samples, while the genus *Phoma* was present exclusively in winter samples (Figure 4B).

### 3.4 Relationship between soil parameters and microbial communities

To address our initial questions about the interplay between soil parameters and microbial communities, regression analyses were conducted to explore correlations between microbial diversity indices and various soil parameters.

#### 3.4.1 Soil physicochemical factors

**Figure 5:**
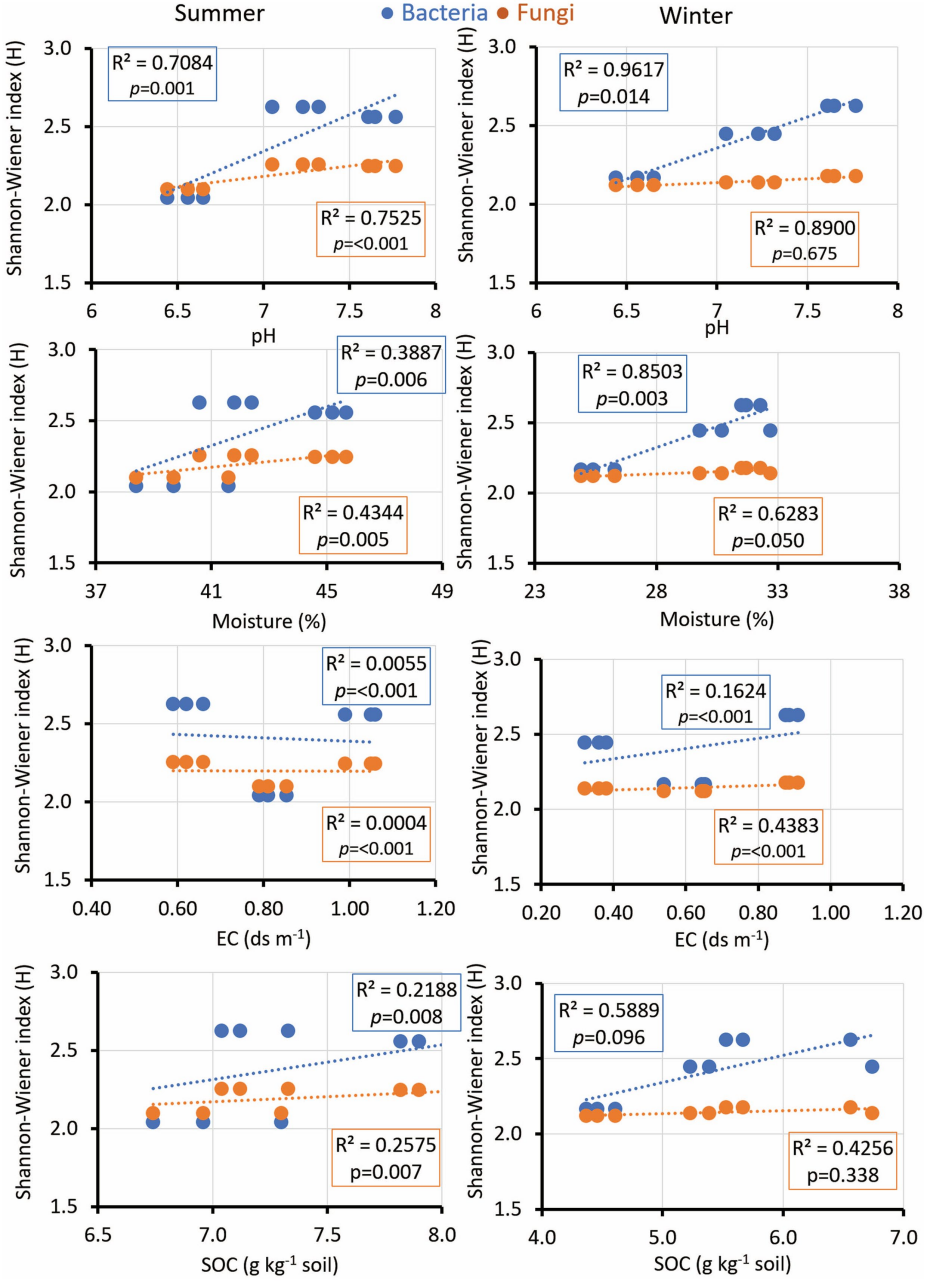
Linear regression relationship between the diversity (Shannon index) of bacteria and fungi versus soil parameters (pH, moisture; EC, electric conductivity; and SOC, soil organic carbon) during two seasons (summer and winter).

**Figure 6:**
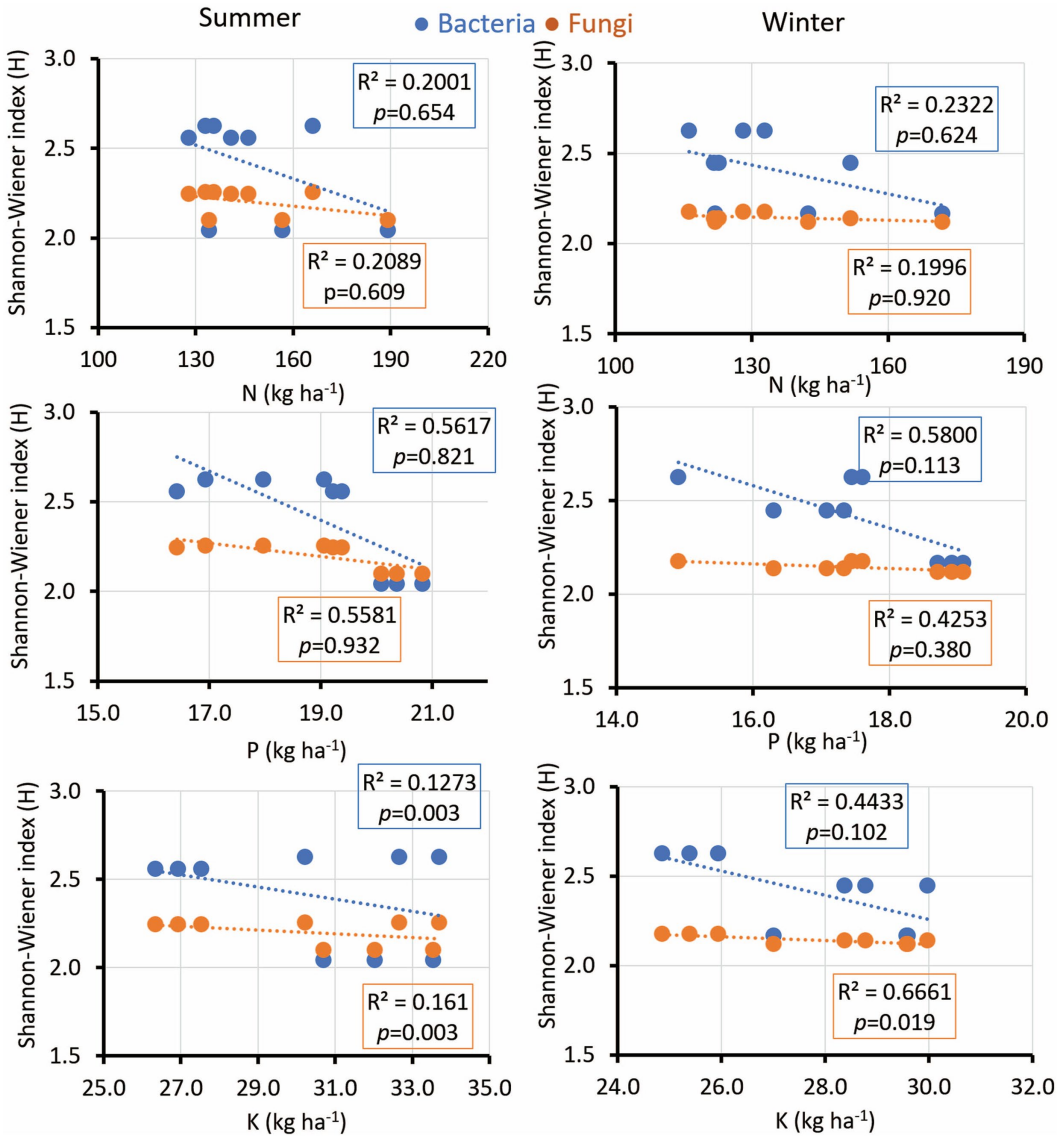
Linear regression relationship between the diversity (Shannon index) of bacteria and fungi versus soil parameters (available N,P, and K) during two seasons (summer and winter).

The microbial diversity indices diversity indices exhibited notable correlations with soil pH, EC, SOC, soil moisture, and nutrient concentrations. Interestingly, the microbial diversity index’s positive correlation with soil pH was consistent across seasons, fostering bacterial growth while suppressing fungal diversity in winter (Figure 5). EC displayed significance across both seasons, while SOC exhibited a comparable trend with a low R-value yet a significant *p*-value during the summer (Figure 5). Soil moisture is positively associated with microbial diversity in both bacterial and fungal communities during winter. Additionally, soil K revealed a significant correlation only with fungal diversity during winter (Figure 6). Notably, no significant association was observed between soil N and P and the diversity indices of isolated fungal and bacterial strains during the study period (Figure 6).

#### 3.4.2 Soil micronutrients and enzyme activities

**Figure 7:**
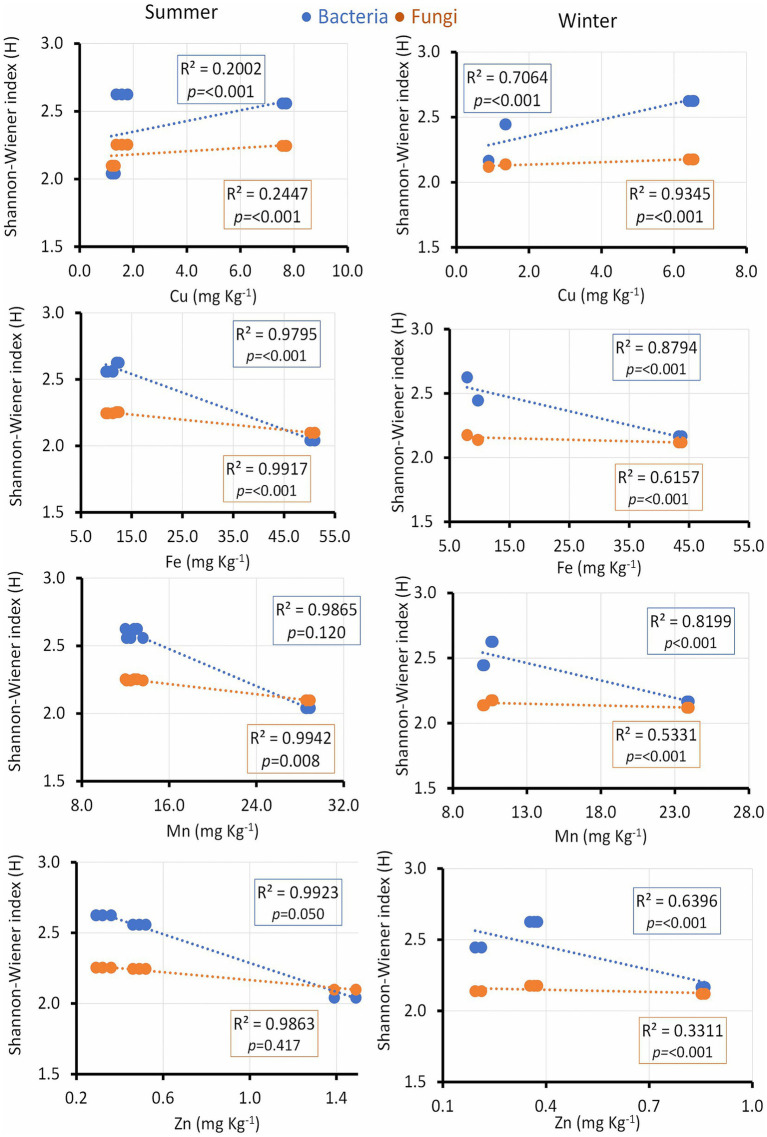
Linear regression relationship between the diversity (Shannon index) of bacteria and fungi versus soil micronutrients (Cu, Fe, Mn and Zn) during two seasons (summer and winter).

Microbial diversity indices were further assessed in relation to soil micronutrients and enzyme activities. Cu exhibited a positive correlation with the microbial diversity index only in winter, while Fe showed a positive correlation in both seasons. Soil Mn positively correlated with the bacterial diversity index in winter and with fungal diversity in summer. Zn showed a positive relationship with the bacterial diversity index in both seasons (Figure 7).

**Figure 8:**
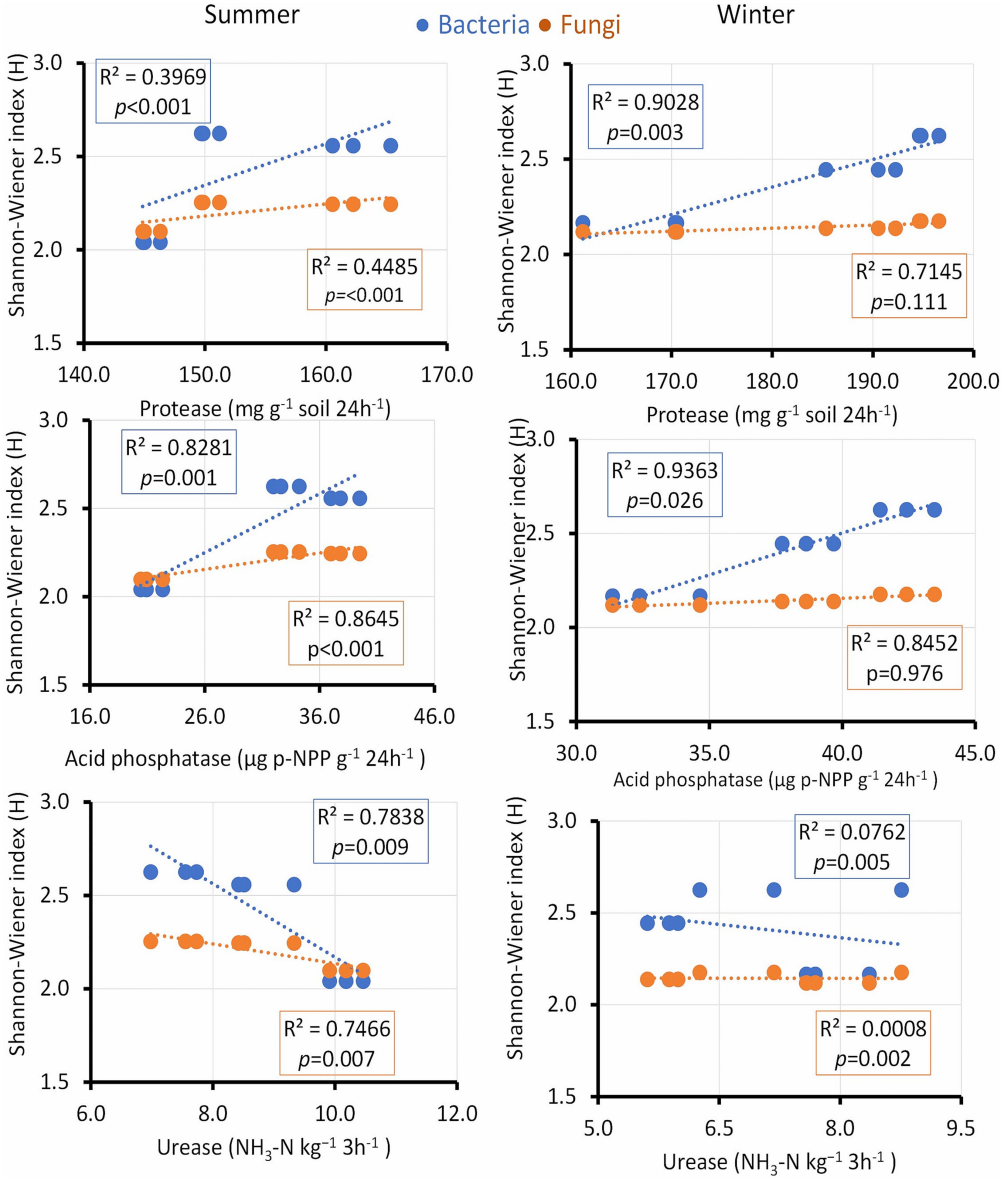
Linear regression relationship between the diversity (Shannon index) of bacteria and fungi versus soil enzymes during two seasons (v).

The bacterial diversity index positively correlated with protease activity in winter, whereas acid phosphatase activity was strongly associated with a microbial diversity shift in summer and was related to bacteria only in winter (Figure 8). Urease activity showed a positive correlation only in summer. Chitinase and nitrate reductase enzymes demonstrated modest R values but maintained a significant *p* value, indicating their noteworthy influence on microbial diversity across both seasons. Additionally, dehydrogenase, characterized by a modest R value, continued to exhibit a significant p value in its association with both bacterial and fungal diversity indices throughout both seasons, underscoring its potential impact on shaping microbial communities (Supplementary Figure S2).

#### 3.4.3 Microbial genera and soil parameters

**Figure 9:**
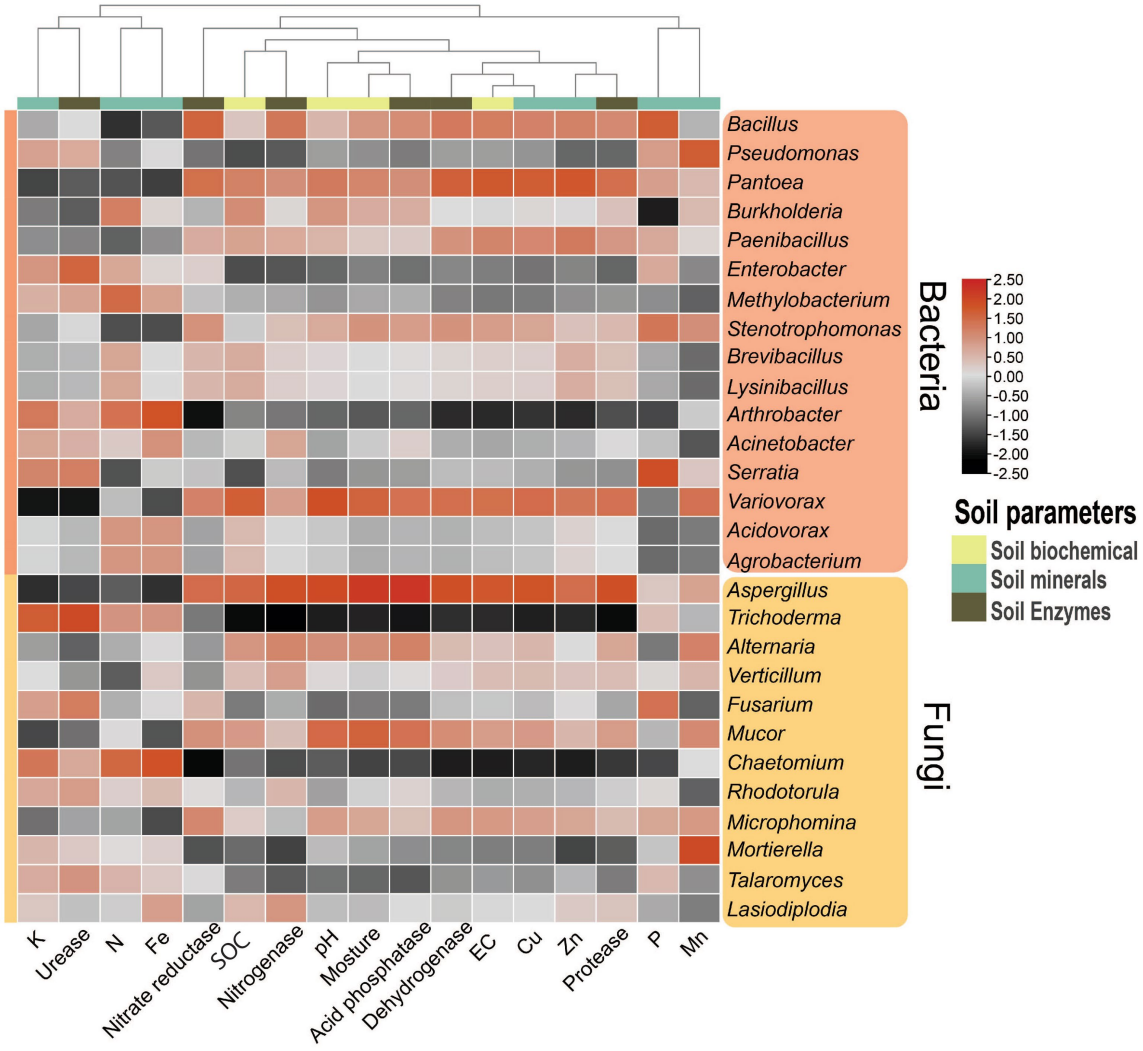
Pearson correlation heatmap showing the relationships between soil parameters (physiological, chemical, mineral, and enzymes) and the dominant genera of bacteria (top 16) and fungi (top 12). EC, electric conductivity; SOC, soil organic carbon.

Regarding specific microbial genera, soil pH significantly (*p* < 0.1) positively correlated with Var*iovorax*, and EC showed a significant (*p* < 0.1) positive correlation with *Pantoea*. The genus *Arthrobacter* exhibited substantial (*p* < 0.1) negative correlations with EC, dehydrogenase, and nitrate reductase activities (Figure 9; Supplementary Table S6). Furthermore, available N showed a significant (*p* < 0.1) positive correlation with *Burkholderia*. Available P exhibited a positive correlation (*p* < 0.05) with *Bacillus* and *Serratia*, while a negative correlation (*p* < 0.05) was found with *Burkholderia*. Additionally, available K showed a significant (*p* < 0.1) negative correlation with the genus *Variovorax*. Genus *Pantoea* demonstrated substantial (*p* < 0.1) positive correlations with Cu, Zn, and dehydrogenase enzyme activity, and the genus *Pseudomonas* showed a significant positive correlation with Mn (Figure 9; Supplementary Table S7).

In the case of fungi, the genus *Aspergillus* significantly (*p* < 0.1) positively correlated with soil pH, moisture, EC, Cu, dehydrogenase, protease, acid phosphatase, and urease activities, while it significantly (*p* < 0.1) negatively correlated with available K and Fe (Figure 9; Supplementary Table S8). The genus *Trichoderma* exhibited a significant (*p* < 0.1) positive correlation with available K and chitinase activity and significantly (*p* < 0.1) negatively correlated with soil pH, moisture, EC, SOC, Cu, dehydrogenase, protease, acid phosphatase, and urease activities. On the other hand, the genus *Chaetomium* showed a significant (*p* < 0.1) positive correlation with N and negatively correlated with EC and nitrate reductase activity (Figure 9).

## 4 Discussion

Plant diversity and element cycling in forest ecosystems depend on the enzymatic activity of the soil. The present investigation is consistent with previous studies that highlight the significance of enzymes, including but not limited to catalase, dehydrogenase, polyphenol oxidase, urease, chitinase, acid-phosphatase, nitrogenase, and invertase, in influencing soil processes and promoting plant diversity (Lladó et al., 2017; Bhaduri et al., 2022; Lull et al., 2023; Zuccarini et al., 2023). The current study showed close relationships between enzyme activities and nutrient availability in soil microbes. These findings suggest that the microbial activity and nutrient availability of tropical forests significantly influence soil enzyme activities (Cai et al., 2018; Harnvoravongchai et al., 2020). Seasonal variations, driven by temperature and precipitation variations, significantly impact soil parameters and microorganism activity, like microbial community composition and function (Bowd et al., 2022). Factors such as pH, moisture content, and nutrient levels strongly influence enzyme activities, ultimately affecting organic matter decomposition and nutrient cycling (Bastida et al., 2021; Shang et al., 2021). The positive correlations between specific microbes and soil properties highlight the interconnected nature of these factors (Žifčáková et al., 2016; Mason et al., 2021). Despite seasonal changes, a stable portion of microbial genera persists, influenced by factors such as root exudates and litter decomposition (Baldrian et al., 2023).

The impacts on forest soils encompass multiple factors, including seasonal variations, natural disasters, and human activities, all of which significantly influence macro and micro-level below-ground processes (Anderson, 2011; Keenan et al., 2015; Halofsky et al., 2020). Our study, conducted in Madhya Pradesh, India, adds valuable insights by investigating the influence of seasonal variations on soil parameters and microbial community structure in dry deciduous forests. The soil of the dry deciduous forests in Madhya Pradesh contains distinct flora, fauna, and environmental conditions that are prominent for studying the forest ecosystem. The selection of these forest sites helps to shed light on the microbial diversity associations with ecological dynamics in this region. The observed diversity in the soil microbial community structure aligns with previous research, indicating the effects of seasonal variation and other biotic and abiotic factors on soil properties (Gray et al., 2015; Waldron et al., 2017; Raha et al., 2020; Han et al., 2023; Liu et al., 2023; Lv et al., 2023).

Soil texture, pH, moisture availability, and nutrient supply emerge as additional parameters significantly impacting both enzyme and microbial activity (Schneider et al., 2008; Cusack et al., 2011; Peña-Claros et al., 2012; Koranda et al., 2013; Chacon et al., 2023; Hartmann and Six, 2023; Wang L. et al., 2023). Soil texture influences water-holding capacity, with acidic soils potentially limiting nutrient availability and impacting microbial activity, while alkaline soils may affect enzymatic reactions. Reduced moisture can constrain microbial activity, affecting nutrient cycling and decomposition of organic matter. Insufficient nitrogen may also limit microbial growth, impacting enzyme production, soil health, and plant diversity. Higher moisture levels in forest soil can lead to a higher SOC, positively affecting microbial diversity (Strukelj et al., 2021; Ren et al., 2022). Tree species diversity and higher moisture levels may enhance SOC and microbial diversity, contributing to biogeochemical stability and nutrient availability (Chen et al., 2018). The organic layer of forest soil, enriched by the decomposition of forest litter, contains a higher concentration of SOC. SOC, in turn, plays a vital role in energy cycling and nutrient transport, including N, P, and K (Allard et al., 2006; Koyama et al., 2018; Farooq et al., 2022). In the current study, soil SOC, N, and P fluctuated less across seasons and locations. Micronutrients such as Fe, Mn, and Zn exhibit were found to be higher in summer, likely influenced by factors such as organic matter addition, root deposition, and microbial activity (Hossain et al., 2011; Naik et al., 2018; Giweta, 2020; Asigbaase et al., 2021). Seasonal changes in soil organic carbon dynamics are associated with changes in Fe levels, which impact the patterns of micronutrients in different areas. As a reflection of the complex interactions in soil ecosystems, more SOC increases Fe availability. In the summer, NV samples showed higher levels of Fe, which were also linked to higher SOC. In both seasons, all micronutrients gradually decreased in all locations, and this was related to the soil pH under different land use systems aligned with previous studies (Edwards and Jefferies, 2013; Dhaliwal et al., 2019).

Forest soil microorganisms play a crucial role in ecological functions by breaking down soil organic matter. Microbial enzymes are vital for decomposing organic materials and acquiring carbon and nutrients. Additionally, soil microbial communities are directly influenced by external environmental changes and play a role in maintaining ecosystem balance. Enzymes catalyzing soil organic matter decomposition play a significant role in nutrient cycling, ecological quality, and agronomic productivity (Alkorta et al., 2003; Sobucki et al., 2021). This enzymatic activity enhances soil texture and contributes to the overall improvement of the soil ecosystem. Our study reveals seasonal variations in enzyme activities, with dehydrogenase, nitrate reductase, and urease activities increasing in summer, while chitinase, protease, and acid phosphatase activities were higher in winter. These findings align with existing literature highlighting the influence of seasons on enzyme activity and microbial processes in healthy soil (Costa et al., 2013; Machmuller et al., 2016; Chen et al., 2018; Kittredge et al., 2018). The correlations observed between pH, moisture, EC, SOC, and enzyme activities further emphasize the interconnected nature of these parameters and their impact on nutrient availability (Han et al., 2021).

Our study reveals a notable predominance of bacteria over fungi. This suggests that bacteria are better adapted to local weather and soil biological processes. This phenomenon indicates the stress-resistant nature of bacteria, contributing significantly to the stability of the soil ecosystem (Gao et al., 2017; Fan et al., 2021). The dominance of specific bacterial genera, including *Bacillus, Pseudomonas, Burkholderia, Pantoea, Paenibacillus, Methylobacterium, Enterobacter, Arthrobacter, Lysinibacillus*, and *Brevibacillus*, highlights their involvement in crucial soil bioprocesses such as phosphorus solubilization and nitrogen mineralization (Patil and Solanki, 2016; Wang et al., 2017, 2023a; Solanki et al., 2019, 2020, 2023; Baldrian et al., 2023; Singh and Dubey, 2023). The dominance of Proteobacteria (genera *Pseudomonas, Burkholderia, Pantoea, Methylobacterium, Enterobacter,* and *Arthrobacter*) and Firmicutes (genera *Bacillus, Paenibacillus, Lysinibacillus*, and *Brevibacillus*) indicates the prevalence of major ligninolytic bacteria commonly found in the rhizosphere and forest soil (Eichorst and Kuske, 2012; Štursová et al., 2012; Urbanová et al., 2015; Lladó et al., 2017). The low dominance of Actinobacteria (genera *Arthrobacter* and *Brevibacterium*) suggests that a higher level of biological activity is stabilized in the forest soil. Our study identifies a seasonal influence on microbial abundance, with fluctuations in response to changing environmental conditions. Interestingly, despite seasonal variations, the microbial community structure remains relatively stable, with a significant overlap in bacterial genera across locations and seasons. This resilience suggests that the forest soil microbiota maintains a consistent structure, reflecting the intricate balance of litter decomposition, nutrient availability, and temperature and precipitation fluctuations (Rasche et al., 2010; Kavitha et al., 2020). The high release of root exudates and leaf litter by forest trees contributes to soil carbon and nitrogen accumulation, further stabilizing soil bacterial communities (Liu et al., 2019; Ma et al., 2022). Organic-rich root exudates that support microbial populations maintain seasonal fluctuations in forest soil. The continuous breakdown of litter also creates an environment that is rich in nutrients, which increases the stability and robustness of microbial genera (Zheng et al., 2019; Wang et al., 2023b; Solanki et al., 2024). The study isolates Ascomycota, Mucoromycota, and Basidiomycota fungi, known for their prevalence in forest ecosystems. Notably, the seasonal variation in the abundance of specific fungal genera, such as *Chaetomium, Verticillium, Mortierella, Aspergillus, Alternaria, Rhodotorula*, and *Mucor*, underscores the adaptive strategies employed by microbial taxa to ensure survival in varying seasonal conditions (Murali, 2013; Delgado et al., 2021; Somen Singh et al., 2021). The stability observed in *Trichoderma* and *Fusarium* across seasons suggests a resilience to seasonal variation.

We investigate the complex interactions among plants, soil, seasons, and microbes in the soil of dry deciduous forests. Vegetation is a primary regulator of changes in soil characteristics, and its abundance affects the variety and composition of the soil bacterial population (Dinakaran et al., 2019). The structure and functions of the soil microbial community are also influenced by the physiological traits of various plant species (Kumar et al., 2019). Enzymes, minerals, and soil characteristics all have significant positive connections with microorganisms (fungi and bacteria), which provide essential information about how these elements are interlinked. For example, the positive correlation between Var*iovorax* and *Aspergillus* with pH, *Aspergillus* with moisture, *Burkholderia* and *Chaetomium* with N, *Bacillus* and *Serratia* with P, and *Trichoderma* with K and chitinase. *Pantoea* and *Aspergillus* were positively correlated with EC, Cu, and dehydrogenase, *Pantoea* with Zn, and *Pseudomonas* with Mn. Conversely, *Arthrobacter* was negatively correlated with EC, dehydrogenase, and nitrate reductase, *Burkholderia* with P, *Variovorax* and *Aspergillus* with K, *Trichoderma* with moisture, EC, SOC, Cu, dehydrogenase, protease, acid phosphatase, and urease, and *Chaetomium* with EC and nitrate reductase. *Aspergillus* was found to adapt well to stress and higher pH conditions, while in winter, soil pH decreased, impacting available K and soil microbial processes (Lladó et al., 2017b; Markina-Iñarrairaegui et al., 2020; Rawal et al., 2022). Soil C, N, and P cycling involve multiple microbial processes such as mineral acquisition, transportation, and availability to plants (Yang et al., 2010; Brockett et al., 2012; Koyama et al., 2018; Liu et al., 2019; Świątek and Pietrzykowski, 2022). The decomposition of litter enriches the organic layer in forest soil, which plays a crucial role in influencing energy cycling, nutrient transport, and the overall functioning of ecosystems. Knowledge about the interactions between SOC, N, P, and micronutrients in the organic layer is essential for comprehending the resilience and efficacy of forest ecosystems. The study shows that the diversity of soil bacteria and fungi is influenced by soil properties, minerals, and enzymes, confirming that location and season impact forest soil fertility. Enzymes and easily accessible minerals are indicators of healthy soil maintained by the structure of the microbial community, and these microorganisms are crucial to soil biological activities(Page-Dumroese et al., 2021). The compositional variation of microbial communities among different locations reflects the adaptability of native communities and the effects of different plant species, weather, and root exudates to some extent (Lauber et al., 2008; Yang et al., 2010; Anderson, 2011; Iwara et al., 2011; Lladó et al., 2017).

## 5 Conclusion

In conclusion, the microbial communities and soil properties in Madhya Pradesh’s dry deciduous forests are significantly impacted by seasonal variations. Soil enzyme activities are strongly correlated with pH, moisture, and nutrients. Soil enzymes are vital catalysts in biogeochemical processes, and the physicochemical environment of the soil influences their activity. The interplay between pH, moisture, EC, SOC, and enzyme activities influences soil nutrient availability, and the delicate balance between these interrelated elements determines how well nutrient cycling processes work, which in turn affects ecosystem productivity and health. Positive relationships between soil characteristics and microbes (bacteria and fungi) highlight the linkages between these two factors. Strong correlations demonstrate how bacteria and fungi interact with minerals, soil properties, and soil enzymes to influence soil fertility. The decomposition of organic matter and nutrient cycling in soil is primarily driven by enzymes, whose activity is influenced by pH, moisture, and SOC. Understanding these relationships is essential to managing ecosystems more intelligently, encouraging sustainability, and improving conservation efforts. Advanced sequencing technologies can be used to study seasonal variations in microbial populations in more depth. These findings might be helpful to in biodiversity conservation and management strategies by examining the diversity of soil microbes in tropical dry deciduous forests. With state-of-the-art sequencing tools, our findings will hopefully be integrated with future studies to increase our efforts to protect these ecosystems by giving us a better knowledge of the complex interactions between bacteria, plants, and seasonal fluctuation in forest soil.

## Data availability statement

The original contributions presented in the study are publicly available. This data can be found at: OP810695–OP810805 (16S rRNA sequence) and OP810826–OP810906 (ITS sequence).

## Author contributions

AS: Conceptualization, Data curation, Visualization, Writing – original draft. NG: Data curation, Supervision, Writing – original draft. SS: Data curation, Supervision, Writing – original draft. ZW: Formal analysis, Funding acquisition, Methodology, Writing – review & editing. AK: Formal analysis, Validation, Writing – review & editing. PK: Formal analysis, Validation, Writing – review & editing. MS: Conceptualization, Visualization, Writing – original draft, Writing – review & editing. KY: Formal analysis, Writing – review & editing. BK: Formal analysis, Writing – review & editing.
