# Supplementary material for: Decoding seasonal changes: soil parameters and microbial communities in tropical dry deciduous forests
Source: Front Microbiol. 2024 Feb 19;15:1258934. doi: 10.3389/fmicb.2024.1258934 (PMC10910104; doi:10.3389/fmicb.2024.1258934)
Supplement: Supplementary file 1 [file Data_Sheet_1.docx]

**Supplementary files**

Table S1: Study area details.

| Code | Village# | longitude | latitude | Average temperature (day/night) | | Tehsil/District |
| --- | --- | --- | --- | --- | --- | --- |
|  |  |  |  | Summer | Winter |  |
| MP | Kotara | 22.88 | 77.08 | 42°C/37°C | 27°C/13°C | Ichhawar/ Sehore |
| NV | Barandua | 22.74 | 77.63 | 40°C/36°C | 25°C/12°C | Hoshangabad/ Hoshangabad |
| SP | Bakud | 22.11 | 78.24 | 43°C/38°C | 28°C/14°C | Ghoradongri/Betul |

#study site situated near this village

Table S2: Diverse environmental conditions of selected sites of three distinct of Madhya Pradesh’s tropical dry deciduous forests.

| Properties | Kotara/Ichhawar/ Sehore | Barandua/Hoshangabad/ Hoshangabad | Bakud/Ghoradongri/Betul |
| --- | --- | --- | --- |
| Soil Fertility | Less fertile soils on the Malwa Plateau. | Fertile alluvial soils | Moderate fertility in hilly areas. |
| Forest Plant Species | Dry deciduous forests are impacted by agriculture. | Mix of dry and moist deciduous forests. | Rich plant diversity. |
| Wildlife | Wildlife habitat affected by land-use changes | Supports diverse wildlife | Known for significant wildlife. |
| Landscaping | Flatter landscape with agriculture. | Features riverine ecosystems. | Diverse with hills, valleys, and forests. |
| Rainfall Patterns | Diverse rainfall patterns | Consistent precipitation from the Narmada River. | Varied rainfall due to topography. |
| Human Interference | Extensive agriculture and urbanization. | Human pressure on riparian forests. | Lower human interference in protected areas. |
| Soil Types | Generally, less fertile soils on the Malwa Plateau. | Fertile alluvial soils. | Varied soil types in the Satpura Range. |

Table S3: Primer and PCR programs used in the study.

| Gene | Primer  name | Sequence (5′-3′) | PCR mix (50 μL) | PCR conditions |
| --- | --- | --- | --- | --- |
| 16Sr RNA | 27F | AGAGTTTGATCMTGGCTCAG | 2X PCR master mix-25 μL, DNA template 1 μL (50 ng), each primer 1 μL (10 μM), and ddH_2_O 22 μL | 95°C-10 min, 35 cycles (90°C-30s, 50°C-30s & 72°C-2 min); 72°C |
|  | 1492R | TACGGYTACCTTGTTACGACTT |  |  |
| ITS | ITS1 | TCCGTAGGTGAACCTGCGG |  | 95°C-3 min, 30 cycles (95°C-30s, 56°C-60s & 72°C-90s); 72°C-5 min |
|  | ITS4 | TCCTCCGCTTATTGATATGC |  |  |

Table S4: Diversity indices for the microbial communities of tropical dry deciduous forest in two seasons

|  | Shannon-Wiener index (H) | | Species richness (S) | | Evenness (E_H_) | |
| --- | --- | --- | --- | --- | --- | --- |
|  | Summer | Winter | Summer | Winter | Summer | Winter |
| **Bacteria** |  |  |  |  |  |  |
| MP | 2.63 | 2.45 | 15.00 | 13.00 | 0.92 | 0.89 |
| NV | 2.56 | 2.63 | 15.00 | 15.00 | 0.86 | 0.92 |
| SP | 2.04 | 2.17 | 9.00 | 10.00 | 0.86 | 0.87 |
| **Fungi** |  |  |  |  |  |  |
| MP | 2.25 | 2.14 | 10.00 | 9.00 | 0.95 | 0.94 |
| NV | 2.25 | 2.18 | 10.00 | 10.00 | 0.94 | 0.88 |
| SP | 2.10 | 2.12 | 9.00 | 9.00 | 0.91 | 0.92 |
| MP-Malva plateau, NV-Narmada valley, and SP-Satpura valley | | | | | | |

Table S5: List of bacterial genera and closely related species with accession no.

| Isolate | Genera | Species | Acc. no. |
| --- | --- | --- | --- |
| MpS01 | *Methylobacterium* | *Methylobacterium zatmanii* | OP810695 |
| MpS02 | *Pantoea* | *Pantoea agglomerans* | OP810696 |
| MpS03 | *Enterobacter* | *Enterobacter sp.* | OP810697 |
| MpS04 | *Pseudomonas* | *Pseudomonas mosselii* | OP810698 |
| MpS05 | *Agrobacterium* | *Agrobacterium rhizogenes* | OP810699 |
| MpS06 | *Acidovorax* | *Acidovorax sp.* | OP810700 |
| MpS07 | *Variovorax* | *Variovorax sp.* | OP810701 |
| MpS08 | *Pseudomonas* | *Pseudomonas aeruginosa* | OP810702 |
| MpS09 | *Bacillus* | *Bacillus licheniformis* | OP810703 |
| MpS10 | *Burkholderia* | *Burkholderia sp.* | OP810704 |
| MpS11 | *Brevibacillus* | *Brevibacillus sp.* | OP810705 |
| MpS12 | *Paenibacillus* | *Paenibacillus sp.* | OP810706 |
| MpS13 | *Lysinibacillus* | *Lysinibacillus fusiformis* | OP810707 |
| MpS14 | *Exiguobacterium* | *Exiguobacterium undae* | OP810708 |
| MpS15 | *Bacillus* | *Bacillus subtilis* | OP810709 |
| MpS16 | *Achromobacter* | *Achromobacter animicus* | OP810710 |
| MpS17 | *Arthrobacter* | *Arthrobacter globiformis* | OP810711 |
| MpS18 | *Burkholderia* | *Burkholderia sp.* | OP810712 |
| MpS19 | *Pseudomonas* | *Pseudomonas fluorescens* | OP810713 |
| MpW01 | *Alcaligenes* | *Alcaligenes faecalis* | OP810714 |
| MpW02 | *Bacillus* | *Bacillus sp.* | OP810715 |
| MpW03 | *Enterobacter* | *Enterobacter cloacae* | OP810716 |
| MpW04 | *Pseudomonas* | *Pseudomonas sp.* | OP810717 |
| MpW05 | *Acinetobacter* | *Acinetobacter sp.* | OP810718 |
| MpW06 | *Serratia* | *Serratia symbiotica* | OP810719 |
| MpW07 | *Pseudomonas* | *Pseudomonas songnenensis* | OP810720 |
| MpW08 | *Stenotrophomonas* | *Stenotrophomonas sp.* | OP810721 |
| MpW09 | *Bacillus* | *Bacillus aryabhattai* | OP810722 |
| MpW10 | *Aeromonas* | *Aeromonas sp.* | OP810723 |
| MpW11 | *Burkholderia* | *Burkholderia cepacia* | OP810724 |
| MpW12 | *Methylobacterium* | *Methylorubrum populi* | OP810725 |
| MpW13 | *Pseudomonas* | *Pseudomonas putida* | OP810726 |
| MpW14 | *Achromobacter* | *Achromobacter animicus* | OP810727 |
| MpW15 | *Bacillus* | *Bacillus funiculus* | OP810728 |
| MpW16 | *Arthrobacter* | *Arthrobacter sp.* | OP810729 |
| MpW17 | *Exiguobacterium* | *Exiguobacterium sp.* | OP810730 |
| NvS01 | *Enterobacter* | *Enterobacter amnigenus* | OP810731 |
| NvS02 | *Serratia* | *Serratia marcescens* | OP810732 |
| NvS03 | *Brevibacillus* | *Brevibacillus laterosporus* | OP810733 |
| NvS04 | *Lysinibacillus* | *Lysinibacillus sphaericus* | OP810734 |
| NvS05 | *Bacillus* | *Bacillus subtilis* | OP810735 |
| NvS06 | *Agrobacterium* | *Agrobacterium rhizogenes* | OP810736 |
| NvS07 | *Pantoea* | *Pantoea ananatis* | OP810737 |
| NvS08 | *Bacillus* | *Bacillus cereus* | OP810738 |
| NvS09 | *Paenibacillus* | *Paenibacillus alginolyticus* | OP810739 |
| NvS10 | *Acinetobacter* | *Acinetobacter pittii* | OP810740 |
| NvS11 | *Pseudomonas* | *Pseudomonas aeruginosa* | OP810741 |
| NvS12 | *Alcaligenes* | *Alcaligenes aquatilis* | OP810742 |
| NvS13 | *Pseudomonas* | *Pseudomonas stutzeri* | OP810743 |
| NvS14 | *Methylobacterium* | *Methylobacterium aminovorans* | OP810744 |
| NvS15 | *Acidovorax* | *Acidovorax delafieldii* | OP810745 |
| NvS16 | *Bacillus* | *Bacillus licheniformis* | OP810746 |
| NvS17 | *Pseudomonas* | *Pseudomonas extremaustralis* | OP810747 |
| NvS18 | *Paenibacillus* | *Paenibacillus amylolyticus* | OP810748 |
| NvS19 | *Bacillus* | *Bacillus pumilus* | OP810749 |
| NvS20 | *Arthrobacter* | *Arthrobacter sp.* | OP810750 |
| NvS21 | *Burkholderia* | *Paraburkholderia sabiae* | OP810751 |
| NvW01 | *Agrobacterium* | *Agrobacterium rhizogenes* | OP810752 |
| NvW02 | *Bacillus* | *Bacillus megaterium* | OP810753 |
| NvW03 | *Bacillus* | *Bacillus safensis* | OP810754 |
| NvW04 | *Variovorax* | *Variovorax sp.* | OP810755 |
| NvW05 | *Pantoea* | *Pantoea agglomerans* | OP810756 |
| NvW06 | *Methylobacterium* | *Methylobacterium sp.* | OP810757 |
| NvW07 | *Burkholderia* | *Burkholderia cepacia* | OP810758 |
| NvW08 | *Pseudomonas* | *Pseudomonas helmanticensis* | OP810759 |
| NvW09 | *Acinetobacter* | *Acinetobacter pittii* | OP810760 |
| NvW10 | *Acidovorax* | *Acidovorax sp.* | OP810761 |
| NvW11 | *Burkholderia* | *Burkholderia zhejiangensis* | OP810762 |
| NvW12 | *Pseudomonas* | *Pseudomonas fluorescens* | OP810763 |
| NvW13 | *Arthrobacter* | *Arthrobacter globiformis* | OP810764 |
| NvW14 | *Bacillus* | *Bacillus cereus* | OP810765 |
| NvW15 | *Paenibacillus* | *Paenibacillus alvei* | OP810766 |
| NvW16 | *Aeromonas* | *Aeromonas sp.* | OP810767 |
| NvW17 | *Brevibacillus* | *Brevibacillus invocatus* | OP810768 |
| NvW18 | *Klebsiella* | *Klebsiella pneumoniae* | OP810769 |
| NvW19 | *Lysinibacillus* | *Lysinibacillus varians* | OP810770 |
| SpS01 | *Pantoea* | *Pantoea agglomerans* | OP810771 |
| SpS02 | *Pseudomonas* | *Pseudomonas fluorescens* | OP810772 |
| SpS03 | *Stenotrophomonas* | *Stenotrophomonas sp.* | OP810773 |
| SpS04 | *Bacillus* | *Bacillus anthracis* | OP810774 |
| SpS05 | *Brevibacterium* | *Brevibacterium epidermidis* | OP810775 |
| SpS06 | *Bacillus* | *Bacillus pumilus* | OP810776 |
| SpS07 | *Pseudomonas* | *Pseudomonas aeruginosa* | OP810777 |
| SpS08 | *Pantoea* | *Pantoea agglomerans* | OP810778 |
| SpS09 | *Variovorax* | *Variovorax sp.* | OP810779 |
| SpS10 | *Pseudomonas* | *Pseudomonas alcalophila* | OP810780 |
| SpS11 | *Paenibacillus* | *Paenibacillus alvei* | OP810781 |
| SpS12 | *Pantoea* | *Pantoea sp.* | OP810782 |
| SpS13 | *Stenotrophomonas* | *Stenotrophomonas rhizophila* | OP810783 |
| SpS14 | *Bacillus* | *Bacillus lehensis* | OP810784 |
| SpS15 | *Burkholderia* | *Burkholderia pseudomultivorans* | OP810785 |
| SpS16 | *Bacillus* | *Bacillus siamensis* | OP810786 |
| SpS17 | *Pseudomonas* | *Pseudomonas trivialis* | OP810787 |
| SpS18 | *Paenibacillus* | *Paenibacillus amylolyticus* | OP810788 |
| SpS19 | *Serratia* | *Serratia marcescens* | OP810789 |
| SpW01 | *Bacillus* | *Bacillus velezensis* | OP810790 |
| SpW02 | *Pantoea* | *Pantoea sp.* | OP810791 |
| SpW03 | *Pseudomonas* | *Pseudomonas alcalophila* | OP810792 |
| SpW04 | *Bacillus* | *Bacillus sp.* | OP810793 |
| SpW05 | *Methylobacterium* | *Methylobacterium populi* | OP810794 |
| SpW06 | *Enterobacter* | *Enterobacter cloacae* | OP810795 |
| SpW07 | *Burkholderia* | *Burkholderia cepacia* | OP810796 |
| SpW08 | *Stenotrophomonas* | *Stenotrophomonas rhizophila* | OP810797 |
| SpW09 | *Lysinibacillus* | *Lysinibacillus sp.* | OP810798 |
| SpW10 | *Bacillus* | *Bacillus subtilis* | OP810799 |
| SpW11 | *Brevibacillus* | *Brevibacillus invocatus* | OP810800 |
| SpW12 | *Enterobacter* | *Enterobacter sp.* | OP810801 |
| SpW13 | *Pantoea* | *Pantoea brenneri* | OP810802 |
| SpW14 | *Paenibacillus* | *Paenibacillus* | OP810803 |
| SpW15 | *Pseudomonas* | *Pseudomonas putida* | OP810804 |
| SpW16 | *Bacillus* | *Bacillus flexus* | OP810805 |

Table S6: List of fungal genera and closely related species with accession no.

| Isolate | Genera | Species | Acc. no. |
| --- | --- | --- | --- |
| FMpS01 | *Talaromyces* | *Talaromyces pinophilus* | OP810826 |
| FMpS02 | *Aspergillus* | *Aspergillus arizonicus* | OP810827 |
| FMpS03 | *Alternaria* | *Alternaria infectoria* | OP810828 |
| FMpS04 | *Mucor* | *Mucor variisporus* | OP810829 |
| FMpS05 | *Fusarium* | *Fusarium proliferatum* | OP810830 |
| FMpS06 | *Chaetomium* | *Chaetomium globosum* | OP810831 |
| FMpS07 | *Mortierella* | *Mortierella alpina* | OP810832 |
| FMpS08 | *Trichoderma* | *Trichoderma rossicum* | OP810833 |
| FMpS09 | *Verticillium* | *Verticillium isaacii* | OP810834 |
| FMpS10 | *Microphomina* | *Macrophomina phaseolina* | OP810835 |
| FMpS11 | *Trichoderma* | *Trichoderma sp.* | OP810836 |
| FMpS12 | *Chaetomium* | *Chaetomium globosum* | OP810837 |
| FMpW01 | *Aspergillus* | *Aspergillus luchuensis* | OP810838 |
| FMpW02 | *Fusarium* | *Fusarium oxysporum* | OP810839 |
| FMpW03 | *Trichoderma* | *Trichoderma reesei* | OP810840 |
| FMpW04 | *Rhodotorula* | *Rhodotorula sp.* | OP810841 |
| FMpW05 | *Alternaria* | *Alternaria sp.* | OP810842 |
| FMpW06 | *Mortierella* | *Mortierella alpina* | OP810843 |
| FMpW07 | *Rhodotorula* | *Rhodotorula glutinis* | OP810844 |
| FMpW08 | *Trichoderma* | *Trichoderma koningiopsis* | OP810845 |
| FMpW09 | *Alternaria* | *Alternaria solani* | OP810846 |
| FMpW10 | *Aspergillus* | *Aspergillus tritici* | OP810847 |
| FMpW11 | *Chaetomium* | *Chaetomium globosum* | OP810848 |
| FMpW12 | *Mucor* | *Mucor plumbeus* | OP810849 |
| FMpW13 | *Verticillum* | *Verticillium zaregamsianum* | OP810850 |
| FNvS01 | *Fusarium* | *Fusarium solani* | OP810851 |
| FNvS02 | *Verticillium* | *Verticillium isaacii* | OP810852 |
| FNvS03 | *Chaetomium* | *Chaetomium globosum* | OP810853 |
| FNvS04 | *Aspergillus* | *Aspergillus tritici* | OP810854 |
| FNvS05 | *Trichoderma* | *Trichoderma erinaceum* | OP810855 |
| FNvS06 | *Lasiodiplodia* | *Diplodia seriata* | OP810856 |
| FNvS07 | *Alternaria* | *Alternaria sp.* | OP810857 |
| FNvS08 | *Verticillium* | *Verticillium zaregamsianum* | OP810858 |
| FNvS09 | *Rhodotorula* | *Rhodotorula graminis* | OP810859 |
| FNvS10 | *Talaromyces* | *Talaromyces pinophilus* | OP810860 |
| FNvS11 | *Trichoderma* | *Trichoderma sp.* | OP810861 |
| FNvS12 | *Leptosphaerulina* | *Leptosphaerulina sp.* | OP810862 |
| FNvS13 | *Fusarium* | *Fusarium oxysporum* | OP810863 |
| FNvW01 | *Trichoderma* | *Trichoderma koningiopsis* | OP810864 |
| FNvW02 | *Fusarium* | *Fusarium proliferatum* | OP810865 |
| FNvW03 | *Verticillum* | *Verticillium tricorpus* | OP810866 |
| FNvW04 | *Chaetomium* | *Chaetomium globosum* | OP810867 |
| FNvW05 | *Aspergillus* | *Aspergillus arizonicus* | OP810868 |
| FNvW06 | *Alternaria* | *Alternaria solani* | OP810869 |
| FNvW07 | *Rhodotorula* | *Rhodotorula glutinis* | OP810870 |
| FNvW08 | *Alternaria* | *Alternaria infectoria* | OP810871 |
| FNvW09 | *Phoma* | *Phoma sp.* | OP810872 |
| FNvW10 | *Mucor* | *Mucor circinelloides* | OP810873 |
| FNvW11 | *Lasiodiplodia* | *Diplodia seriata* | OP810874 |
| FNvW12 | *Aspergillus* | *Aspergillus tubingensis* | OP810875 |
| FNvW13 | *Verticillum* | *Verticillium klebahnii* | OP810876 |
| FNvW14 | *Aspergillus* | *Aspergillus niger* | OP810877 |
| FNvW15 | *Alternaria* | *Alternaria poaceicola* | OP810878 |
| FSpS01 | *Alternaria* | *Alternaria sp.* | OP810879 |
| FSpS02 | *Aspergillus* | *Aspergillus niger* | OP810880 |
| FSpS03 | *Verticillum* | *Verticillium zaregamsianum* | OP810881 |
| FSpS04 | *Trichoderma* | *Trichoderma koningii* | OP810882 |
| FSpS05 | *Mortierella* | *Mortierella alpina* | OP810883 |
| FSpS06 | *Aspergillus* | *Aspergillus luchuensis* | OP810884 |
| FSpS07 | *Verticillum* | *Verticillium tricorpus* | OP810885 |
| FSpS08 | *Microphomina* | *Macrophomina phaseolina* | OP810886 |
| FSpS09 | *Fusarium* | *Fusarium solani* | OP810887 |
| FSpS10 | *Mucor* | *Mucor plumbeus* | OP810888 |
| FSpS11 | *Aspergillus* | *Aspergillus arizonicus* | OP810889 |
| FSpS12 | *Alternaria* | *Alternaria japonica* | OP810890 |
| FSpS13 | *Pichia* | *Pichia kudriavzevii* | OP810891 |
| FSpW01 | *Mucor* | *Mucor brunneogriseus* | OP810892 |
| FSpW02 | *Verticillum* | *Verticillium tricorpus* | OP810893 |
| FSpW03 | *Fusarium* | *Fusarium solani* | OP810894 |
| FSpW04 | *Aspergillus* | *Aspergillus tritici* | OP810895 |
| FSpW05 | *Trichoderma* | *Trichoderma virens* | OP810896 |
| FSpW06 | *Alternaria* | *Alternaria solani* | OP810897 |
| FSpW07 | *Microphomina* | *Macrophomina phaseolina* | OP810898 |
| FSpW08 | *Rhodotorula* | *Rhodotorula sp.* | OP810899 |
| FSpW09 | *Mucor* | *Mucor racemosus* | OP810900 |
| FSpW10 | *Fusarium* | *Fusarium oxysporum* | OP810901 |
| FSpW11 | *Microphomina* | *Macrophomina phaseolina* | OP810902 |
| FSpW12 | *Aspergillus* | *Aspergillus luchuensis* | OP810903 |
| FSpW13 | *Trichoderma* | *Trichoderma rossicum* | OP810904 |
| FSpW14 | *Talaromyces* | *Talaromyces pinophilus* | OP810905 |
| FSpW15 | *Aspergillus* | *Aspergillus niger* | OP810906 |

Table S7: *P* value of correlation between bacterial genera and soil parameters (highlighted values showing significance <0.1)

|  | *Bacillus* | *Pseudomonas* | *Pantoea* | *Burkholderia* | *Methylobacterium* | *Arthrobacter* | *Serratia* | *Variovorax* |
| --- | --- | --- | --- | --- | --- | --- | --- | --- |
| pH | 0.677 | 0.595 | 0.246 | 0.433 | 0.546 | 0.293 | 0.488 | 0.097 |
| Moisture | 0.444 | 0.539 | 0.337 | 0.600 | 0.678 | 0.256 | 0.637 | 0.219 |
| EC | 0.230 | 0.628 | 0.065 | 0.997 | 0.404 | 0.076 | 0.846 | 0.235 |
| SOM | 0.758 | 0.251 | 0.276 | 0.347 | 0.782 | 0.535 | 0.344 | 0.167 |
| N | **0.083** | **0.477** | **0.211** | **0.145** | **0.057** | **0.084** | **0.285** | **0.903** |
| P | 0.055 | 0.426 | 0.450 | 0.015 | 0.467 | 0.116 | 0.044 | 0.454 |
| K | 0.678 | 0.502 | 0.151 | 0.410 | 0.626 | 0.238 | 0.383 | 0.050 |
| Cu | 0.275 | 0.577 | 0.085 | 0.907 | 0.431 | 0.110 | 0.779 | 0.221 |
| Fe | 0.190 | 0.987 | 0.096 | 0.851 | 0.433 | 0.010 | 0.928 | 0.213 |
| Mn | 0.549 | 0.094 | 0.820 | 0.804 | 0.109 | 0.727 | 0.945 | 0.290 |
| Zn | 0.248 | 0.384 | 0.063 | 0.937 | 0.614 | 0.131 | 0.704 | 0.273 |
| Chitinase | 0.953 | 0.543 | 0.255 | 0.267 | 0.490 | 0.556 | 0.330 | 0.056 |
| Dehydrogenase | 0.223 | 0.624 | 0.098 | 0.965 | 0.456 | 0.078 | 0.844 | 0.234 |
| Protease | 0.345 | 0.365 | 0.188 | 0.739 | 0.635 | 0.254 | 0.650 | 0.274 |
| Acid phosphatase | 0.408 | 0.449 | 0.416 | 0.637 | 0.731 | 0.342 | 0.679 | 0.318 |
| Nitrate reductase | 0.112 | 0.479 | 0.163 | 0.838 | 0.934 | 0.060 | 0.950 | 0.344 |
| Urease | 0.274 | 0.318 | 0.412 | 0.919 | 0.720 | 0.454 | 0.851 | 0.575 |

Table S8: *P* value of correlation between fungal genera and soil parameters (highlighted values showing significance <0.1)

|  | *Aspergillus* | *Trichoderma* | *Chaetomium* | *Mortierella* |
| --- | --- | --- | --- | --- |
| pH | 0.037 | 0.045 | 0.250 | 0.784 |
| Moisture | 0.011 | 0.074 | 0.172 | 0.659 |
| EC | 0.059 | 0.078 | 0.048 | 0.442 |
| SOC | 0.143 | 0.032 | 0.448 | 0.387 |
| N | 0.265 | 0.281 | 0.044 | 0.857 |
| P | 0.798 | 0.716 | 0.115 | 0.840 |
| K | 0.078 | 0.091 | 0.234 | 0.668 |
| Cu | 0.052 | 0.056 | 0.071 | 0.439 |
| Fe | 0.054 | 0.333 | 0.010 | 0.806 |
| Mn | 0.594 | 0.565 | 0.852 | 0.025 |
| Zn | 0.147 | 0.124 | 0.097 | 0.226 |
| Chitinase | 0.169 | 0.019 | 0.533 | 0.789 |
| Dehydrogenase | 0.035 | 0.085 | 0.045 | 0.478 |
| Protease | 0.058 | 0.039 | 0.164 | 0.321 |
| Acid phosphatase | 0.012 | 0.050 | 0.209 | 0.535 |
| Nitrate reductase | 0.149 | 0.505 | 0.042 | 0.301 |
| Urease | 0.078 | 0.031 | 0.255 | 0.220 |


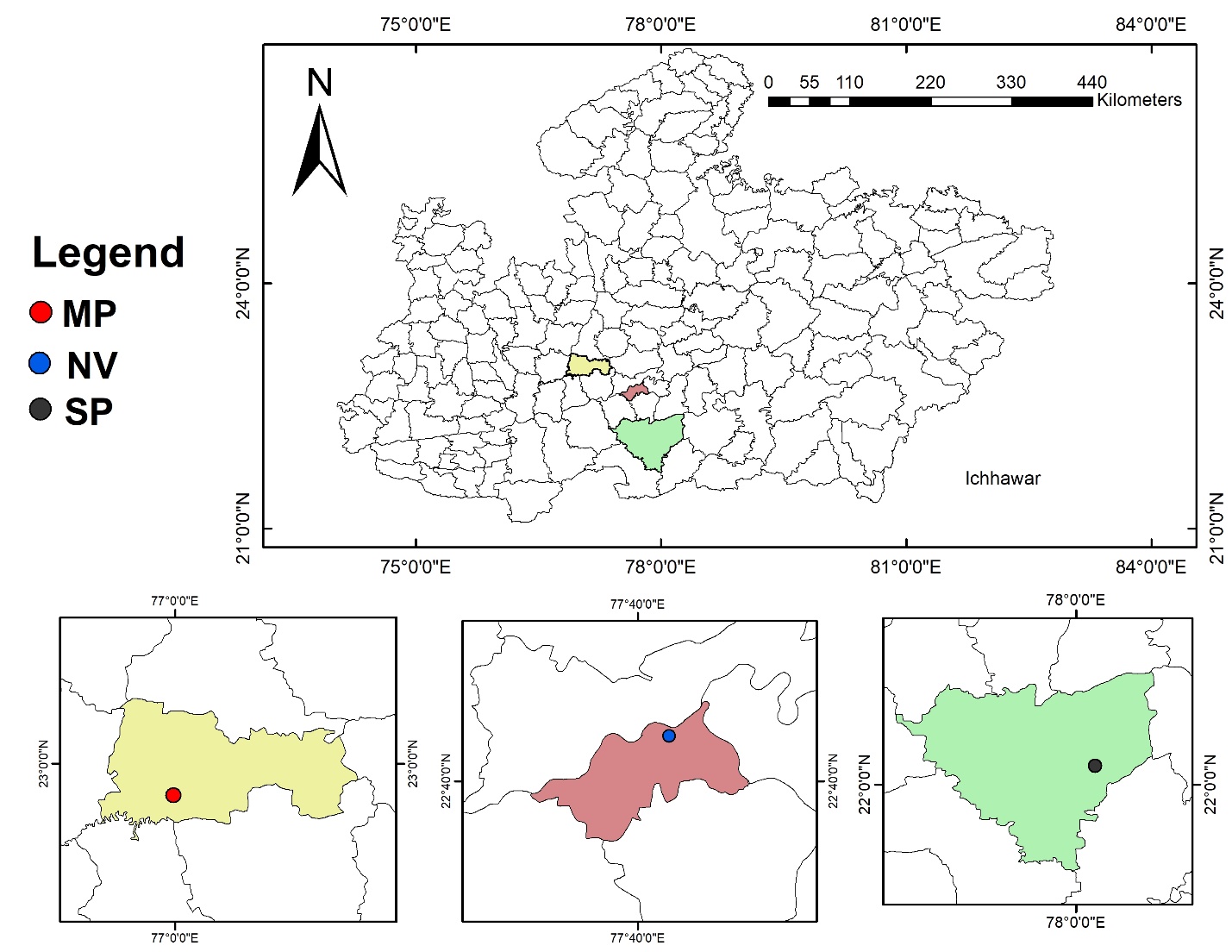


**Supplementary Figure 1**: Location of the study area in three different districts of Madhya Pradesh: MP (Malva plateau), NV (Narmada valley), and SP (Satpura valley).


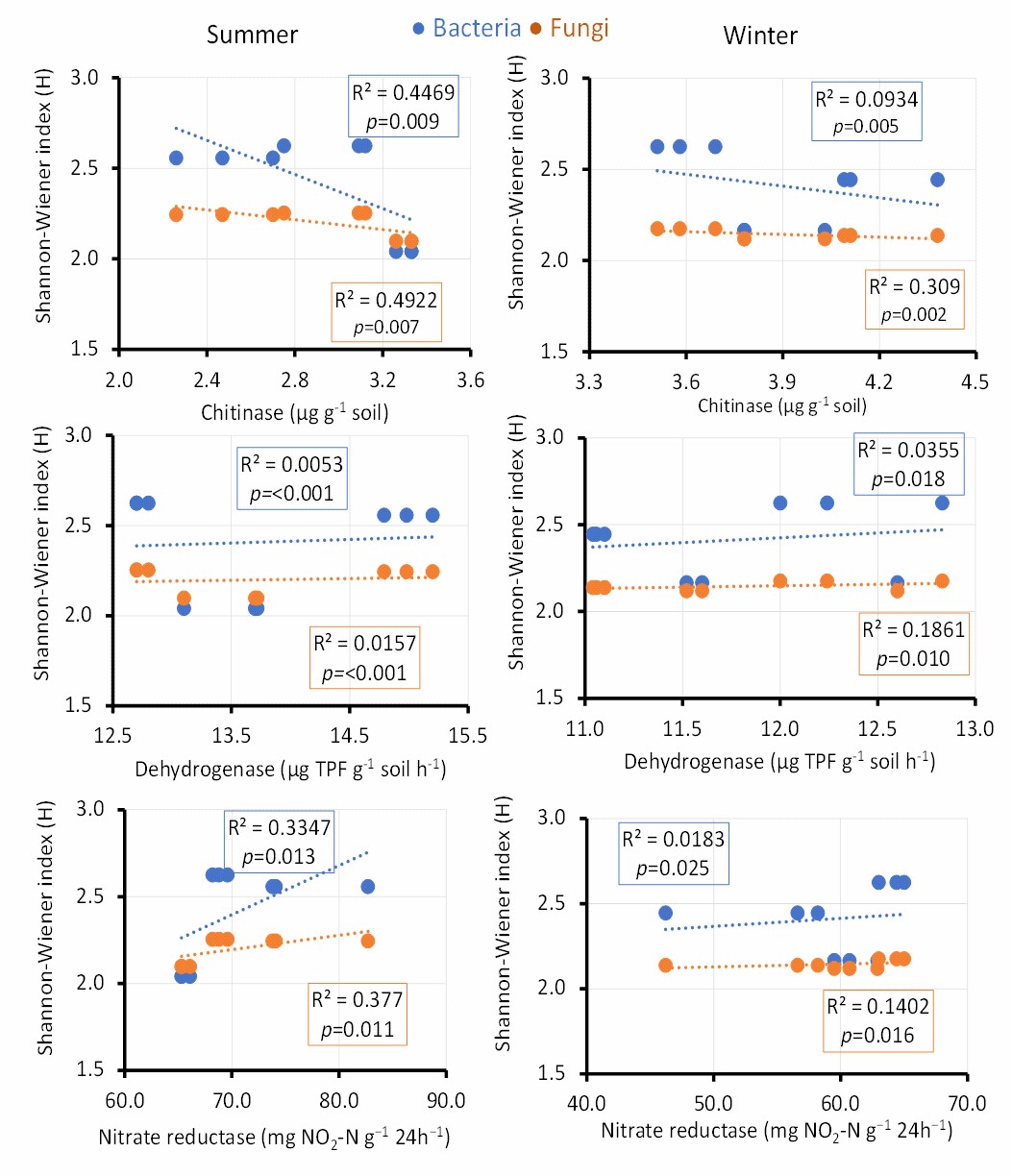


**Supplementary Figure 2**: Linear regression relationship between diversity (Shannon index) of bacteria and fungi versus soil enzymes in two seasons (winter and summer).
